# Oncolytic virus expressing PD-1 inhibitors activates a collaborative intratumoral immune response to control tumor and synergizes with CTLA-4 or TIM-3 blockade

**DOI:** 10.1136/jitc-2022-004762

**Published:** 2022-06-10

**Authors:** Fei Ju, Yong Luo, Chaolong Lin, Xian Jia, Zilong Xu, Rui Tian, Yanhua Lin, Min Zhao, Yating Chang, Xiaoxuan Huang, Shaopeng Li, Wenfeng Ren, Yaning Qin, Mengqin Yu, Jizong Jia, Jinle Han, Wenxin Luo, Jun Zhang, Guo Fu, Xiangzhong Ye, Chenghao Huang, Ningshao Xia

**Affiliations:** 1 State Key Laboratory of Molecular Vaccinology and Molecular Diagnostics, National Institute of Diagnostics and Vaccine Development in Infectious Diseases, School of Public Health, Xiamen University, Xiamen, Fujian, China; 2 Yangshengtang Co., Ltd, Hangzhou, China; 3 Hangzhou Yangshengtang Biopharmaceutical Co., Ltd, Hangzhou, China; 4 School of Life Sciences, Xiamen University, Xiamen, Fujian, China; 5 Beijing Wantai Biological Pharmacy, Beijing, China; 6 School of Medicine, Xiamen University, Xiamen, Fujian, China

**Keywords:** Oncolytic Virotherapy, Tumor Microenvironment, Immunotherapy, Oncolytic Viruses

## Abstract

**Background:**

Oncolytic viruses (OVs) are capable to inflame the tumor microenvironment (TME) and elicit infiltrating tumor-specific T cell responses. However, OV treatment negatively alters the cancer-immune set point in tumors to attenuate the antitumor immune response, which suggests the necessity of dissecting the immune landscape of the virus-treated tumors and developing novel strategies to maximize the potential of OVs. The aim of this study is to investigate the effect of the single-chain variable fragment (scFv)-armed OVs targeting PD-1 on the TME, and ultimately overcome localized immunosuppression to sensitize tumors to immunotherapies.

**Methods:**

A tumor-selective oncolytic herpes simplex virus vector was engineered to encode a humanized scFv against human PD-1 (hPD-1scFv) (YST-OVH). The antitumor efficacy of YST-OVH was explored in multiple therapeutic mouse models. The neurotoxicity and safety of YST-OVH were evaluated in nonhuman primates. The precise dynamics in the TME involved in YST-OVH treatment were dissected using cytometry by time-of-flight (CyTOF).

**Results:**

The identified hPD-1scFv showed superior T-cell activating activity. Localized delivery of hPD-1scFv by YST-OVH promotes systemic antitumor immunity in humanized PD-1 mouse models of established cancer. Immune profiling of tumors using CyTOF revealed the enhanced antitumor effect of YST-OVH, which largely relied on CD8^+^ T cell activity by augmenting the tumor infiltration of effector CD8^+^ T cells and establishment of memory CD8^+^ T cells and reducing associated CD8^+^ T cell exhaustion. Furthermore, YST-OVH treatment modified the cancer-immune set point of tumors coupled to coexpression of CTLA-4 and TIM-3 on exhausted CD8^+^ T cells and high levels of CTLA-4^+^ Treg cells. A combination approach incorporating anti-CTLA-4 or anti-TIM-3 further improved efficacy by increasing tumor immunogenicity and activating antitumor adaptive immune responses. Moreover, this therapeutic strategy showed no neurotoxicity and was well tolerated in nonhuman primates. The benefit of intratumoral hPD-1scFv expression was also observed in humanized mice bearing human cancer cells.

**Conclusion:**

Localized delivery of PD-1 inhibitors by engineered YST-OVH was a highly effective and safe strategy for cancer immunotherapy. YST-OVH also synergized with CTLA-4 or TIM-3 blockade to enhance the immune response to cancer. These data provide a strong rationale for further clinical evaluation of this novel therapeutic approach.

**WHAT IS ALREADY KNOWN ON THIS TOPIC**

- Oncolytic virotherapy using single-chain variable fragment (scFv)-armed oncolytic viruses (OVs) targeting inhibitory immune checkpoints was an effective strategy for cancer immunotherapy. While this proof of concept is demonstrated in mice, studies on clinically relevant models or nonhuman primates are scarce and the cancer-immune set point mediating its effectiveness remains largely unknown.

**WHAT THIS STUDY ADDS**

- Our results demonstrate the potential of a recombinant herpes simplex virus type 1 vector expressing hPD-1scFv as a safe and effective treatment that can promote systemic antitumor immunity by augmenting the effector and memory CD8^+^ T cells and reducing the recruitment of granulocytic myeloid-derived suppressor cells, and overcome localized immunosuppression to sensitize tumors to CTLA-4 or TIM-3 blockade.

**HOW THIS STUDY MIGHT AFFECT RESEARCH**

- The findings will facilitate the understanding of the mechanism of action of an OV expressing humanized hPD-1 inhibitors and encourage the clinical translation of YST-OVH virotherapy.

## Introduction

Oncolytic viruses (OVs) have shown inspiring clinical efficacy in cancer treatments, such as talimogene laherparepvec (Imlygic) in advanced melanoma treatment and oncolytic poliovirus (PVSRIPO) in recurrent glioblastoma treatment.^1 2^ However, clinical benefit has been limited to subsets of patients within a few cancer types. To increase efficacy and extend it to a larger proportion of patients, two critical barriers still need to be overcome.^3^ First, the intratumoral spread of OVs is limited by tumor heterogeneity and the host antiviral immune response, and efficient viral replication and spread within tumors requires more robust OVs. Second, the tumor microenvironment (TME) often contains immunosuppressive factors, and reversing this negative cancer-immune set point requires more appropriate combinations of immunomodulatory agents to induce consistent, durable therapeutic responses against a broad spectrum of cancers.^4^ Our previous study showed that enhancing cell-cell fusion efficiency with a rational designed oncolytic herpes vector could promote cell killing and thereby improve OV efficacy.^5^ Targeting inhibitory immune checkpoints with immunotherapy has produced durable responses in a few subsets of patients with a high tumor mutational burden.^6–9^ Numerous studies have demonstrated that OV treatment significantly improves the efficacy of systemic immune checkpoint blockade in melanoma, colon cancer, etc, including tumor types unresponsive to immune checkpoint blockade.^10–13^

OVs present an attractive engineering platform for combination immunotherapy for two reasons.^14 15^ First, intratumoral inoculation of OVs leads to immune cell infiltration and activation, immunogenic cell death, and antigen release and presentation, which facilitate remodeling of the TME and the induction of tumor antigen-specific antitumor immunity. Second, localized delivery of immunomodulatory agents by OVs offers a myriad of opportunities to target specific pathways directly within tumors, thus potentially avoiding systemic toxicity. Therefore, it is critical to rationally design engineered OVs to selectively replicate within and destroy tumors cells while simultaneously expressing immunomodulatory agents to augment antitumor immunity. OVs can be successfully engineered with immune modulators, such as cytokines, chemokines, costimulatory molecules, and immune checkpoint molecule-specific antibodies, to reshape the immunosuppressive TME and achieve a better antitumor response.^11 16–22^ To this end, we focused on the immune inhibitory signaling mediated through programmed cell death 1 (PD-1), an inhibitory receptor on lymphocytes, which is used by tumors to escape T cell responses through the inhibitory ligand programmed death ligand 1 (PD-L1).^6^ Our previous study showed that the ligand PD-L1 on cancer cells were upregulated in response to intratumoral oncolytic virotherapy, and could be targeted directly within the TME using murine PD-1 blockers expressed by the virus.^22^ OVH-aMPD-1, which was engineered to encode a single-chain variable fragment (scFv) against mouse PD-1 (mPD-1), exhibited robust antitumor activity and prolonged the survival of tumor-bearing mice. However, OVH-aMPD-1 containing an mPD-1 blocker served as a surrogate OV equipped with a PD-1 blocker against human PD-1 (hPD-1), which was destined for human use. There are no reports on the efficacy and safety of an OV armed with a humanized antibody that recognizes hPD-1 in clinically relevant models or nonhuman primates.

Beyond PD-1, there are multiple other immunosuppressive pathways involved in the TME. Immune checkpoint molecules, such as cytotoxic T lymphocyte antigen-4 (CTLA-4), T-cell immunoglobulin and mucin domain-containing protein-3 (TIM-3), T-cell immunoreceptor with immunoglobulin and ITIM domains (TIGIT) and lymphocyte activation gene 3 (LAG-3) are frequently overexpressed on tumor-infiltrating lymphocytes (TILs), leading to the exhaustion of activated CD8^+^ T cells and contributing to the establishment of the cancer-immune set point.^23^ For these reasons, the combination of OVs with immune checkpoint blockade has been considered a promising immunotherapeutic strategy that could further enhance T cell effector function within the TME and improve the antitumor immune response.^24^ However, how the landscape of the TME is reshaped and how T cells are rejuvenated through intratumoral oncolytic virotherapy remain elusive, and which reasonable combinations of OVs and immune checkpoint blockade agents will produce optimal therapeutic effects has yet to be determined.^15^

In this study, we generated an oncolytic herpes simplex virus type 1 (HSV-1) virus, YST-OVH, by inserting a humanized hPD-1 blocker gene, encoding an scFv against hPD-1 (hPD-1scFv), into the parental OVH genome. We then tested the antitumor efficacy of YST-OVH in multiple therapeutic models and a humanized mouse model of established human cancer and evaluated the neurotoxicity and safety of YST-OVH in nonhuman primates. We used cytometry by time-of-flight (CyTOF) to dissect the precise dynamics in the TME and the underlying antitumor mechanism involved in YST-OVH treatment. We also investigated the efficacy of combined application of YST-OVH with CTLA-4 or TIM-3 blockade. The study results thus provide a strong rationale for further evaluation of this novel OV in the clinic.

## Materials and methods

### Mice

Female C57BL/6, BALB/c mice and BALB/c nu/nu mice were purchased from Beijing Vital River Laboratory (Beijing, China). Female C57BL/6J-*Pdcd1* ^em1(hPDCD1)^ and BALB/cJ-*Pdcd1* ^em1(hPDCD1)^ mice (PD-1-HU), in which the endogenous *PDCD1* gene is replaced by a chimeric PD-1 with a human extracellular domain (ECD), a murine transmembrane domain and a murine intracellular domain, were purchased from Shanghai Model Organisms Center (Shanghai, China) and GemPharmatech (Jiangsu, China), respectively. The mice used in this study were 6–8 weeks old unless otherwise indicated

### Virus generation and titration

The full-length hPD-1scFv sequence under the control of the human cytomegalovirus promoter was inserted into the SmaI-digested donor plasmid d34.5/0. The generation of recombinant virus was performed using a cell-based recombination method described previously.^22^ YST-OVH was constructed on the backbone of the OV, OVH, which was developed previously in our laboratory.^25^ YST-OVH is an ICP0- and ICP34.5-null HSV-1 virus, in which both copies of the ICP34.5 and ICP0 coding sequences were replaced by the hPD-1scFv genes and the essential gene ICP27 is under the regulation of the tumor-specific hTERT promoter. The titers of amplified viruses were determined on U-2 OS monolayers using a classical plaque assay.

### Tumor models

For human xenograft tumor models, 5×10^6^ human cancer cells in 100 µL of Phosphate Buffered Saline (PBS) were subcutaneously inoculated into the flanks of BALB/c nu/nu mice. Once the tumors reached 100–200 mm^3^, virus was intratumorally injected at a dose of 10^7^ plaque-forming units (PFU) and then repeatedly injected on days 3 and 6 after the initial treatment.

For murine syngeneic tumor models, 5×10^6^ tumor cells in 100 µL of PBS were subcutaneously inoculated into the right flank or both flanks of C57BL/6 mice or PD-1-HU mice and allowed to establish for 7–12 days. Once the tumors reached the indicated volume, virus was intratumorally injected at a dose of 10^7^ PFU and then repeatedly injected on day 3 and 6 after the initial treatment.

For murine orthotopic tumor models, 5×10^5^ Hepa1-6-luc cells were inoculated into the liver parenchyma of PD-1-HU mice in 50 µL of sterile PBS and allowed to establish for 4 days. Virus was administered by intravenous injection at a dose of 3×10^7^ PFU every 3 days for four consecutive injections. The overall survival of the mice was monitored over an 86-day period. Bioluminescence imaging data was collected using an IVIS Imaging System (Xenogen), every week for 5 weeks after the initial treatment.

For combination therapy, 5×10^6^ tumor cells in 100 µL of PBS were subcutaneously inoculated into the right flank or both flanks of PD-1-HU mice and allowed to establish for 12–15 days. Once the tumors reached the indicated volume, 10^7^ PFU of virus and 200 µg of indicated checkpoint antibodies were intratumorally injected and then repeatedly injected on day 3 and 6 after the initial treatment.

For humanized mouse xenograft models, cord blood CD34^+^ stem cells were obtained from Lonza. Briefly, 3–4-week-old female NSG mice were subjected to 200 cGy total body irradiation 12 hours before tail vein injection of 2×10^5^ cord blood CD34^+^ stem cells in 0.2 mL of medium. Twelve weeks after engraftment, humanized mice with over 25% human CD45^+^ cell reconstitution were used for the tumor study, and 2×10^6^ Huh-7 cells were subcutaneously injected into the right flank of the mice. Beginning 14–16 days after tumor inoculation, mice were injected intratumorally with PBS or YST-OVH every 3 days for 9 days. Tumor growth was evaluated by monitoring tumor volume every 3 days. Animals were sacrificed when the xenograft volume reached ~1000 mm^3^.

For the tumor rechallenge experiment, cured mice and naïve mice were subcutaneously challenged in the alternate flank with 5×10^7^ tumor cells, and the overall survival of the mice was monitored over a 100-day period.

Tumor volume was measured every 3 days for 30 days using calipers and calculated by using the formula Volume=(L×(W)^2^ /2, where L and W represent the largest and the smallest diameters, respectively. The tumor-free incidence is presented as the percentage of tumor-free mice among the total treated mice.

### Statistics

Statistical analyses were performed using GraphPad Prism V.7 software (GraphPad Software). The procedures for comparisons and the numbers of animals in the experiment are described in each figure legend. Differences were considered significant when the P value was less than 0.05. ^*^P<0.05; ^**^P<0.01; ^***^P<0.001, ^****^P<0.0001; ns, not significant.

## Results

### Oncolytic efficacy is restricted by upregulation of PD-1 on tumor-infiltrating T cells

**Figure 1:**
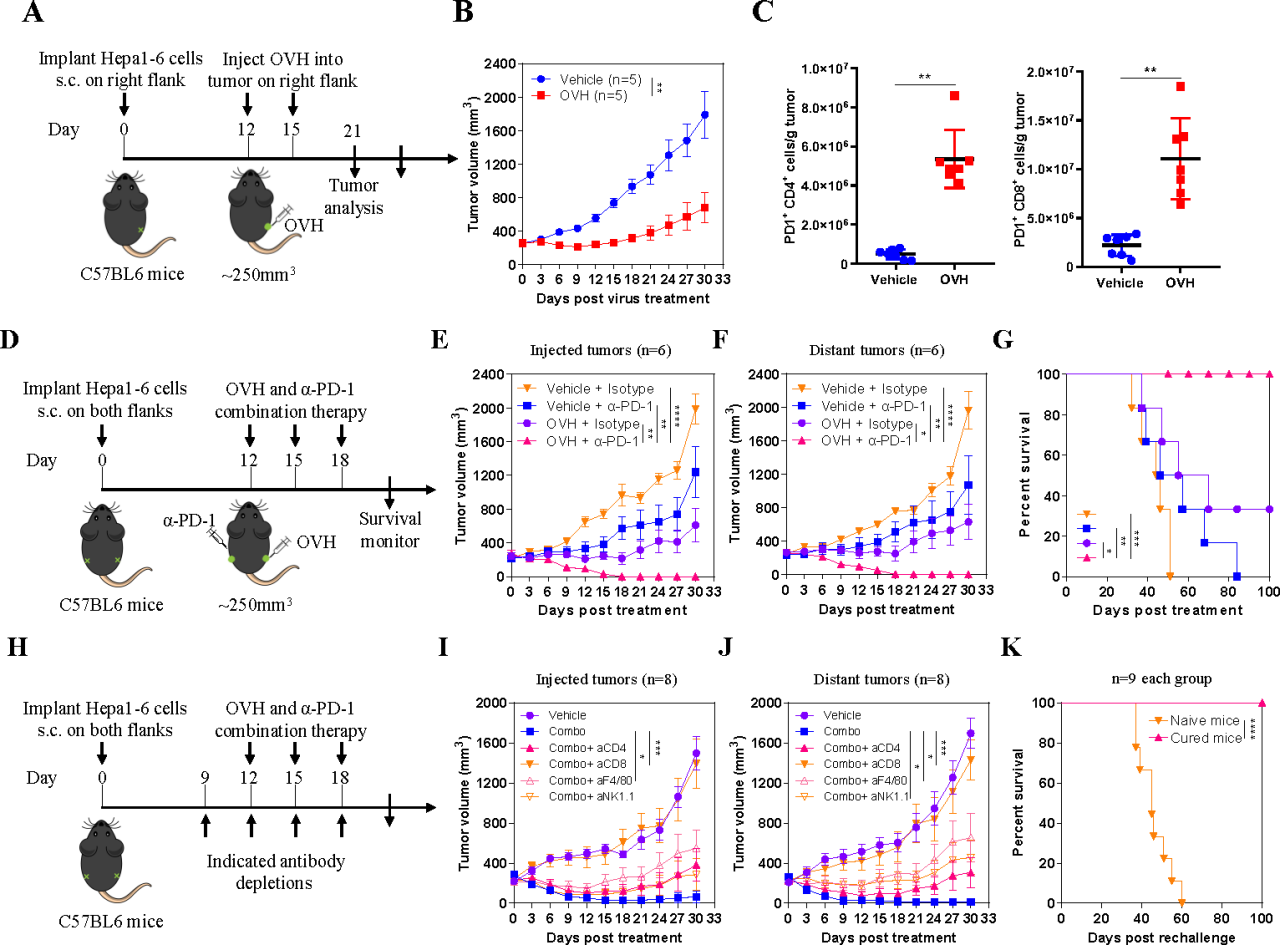
PD-1 upregulation in the tumor microenvironment (TME) restricts the antitumor responses of OVH. (A) Treatment scheme for a syngeneic Hepa1-6 tumor model. (B) Growth of vehicle (PBS)-treated, and OVH-treated Hepa1-6 tumors. (C) Absolute numbers of tumor-infiltrating PD-1^+^CD4^+^ and PD-1^+^CD8^+^ lymphocytes isolated from vehicle-treated and virus-treated tumors. (D) Treatment scheme for bilateral flank Hepa1-6 tumor-bearing mice. Mice bearing tumors received monotherapy or combination therapy. Growth of injected tumors (E) and distant tumors (F). (G) Overall survival was monitored over a 100-day period. (H) Treatment scheme for depletion experiments. Mice bearing Hepa1-6 tumors received combination therapy and the indicated depleting antibodies or isotype antibodies. Growth of injected tumors (I) and distant tumors (J). (K) Survival of mice cured by OVH therapy and rechallenged with 5×10^7^ Hepa1-6 cells. Data represent results from one of three (B, E, F, G) or one of two (C, I–K) independent experiments with n=5 to n=8 per group. Data for survival were analyzed by the log-rank (Mantel-Cox) test (G, K). All values are presented as the mean±SEM; repeated-measure ANOVA (B, E, F, I, J) or unpaired two-tailed Student’s t-test (C). *p<0.05; **p<0.01; ***p<0.001; ****p<0.0001. OVH, oncolytic herpes simplex virus-1; ANOVA, analysis of variance.

In our previous study, we developed an immunotherapeutic oncolytic HSV-1 virus (OVH) that showed potent and durable antitumor effects.^25^ To investigate to what extent large advanced tumors affect oncolytic efficacy, we evaluated the antitumor efficacy of OVH in a syngeneic Hepa1-6 tumor model bearing 250 mm^3^ tumors (figure 1A). OVH therapy significantly inhibited tumor growth but did not completely eradicate the tumors (figure 1B). While OVH remodeled the TME by enhancing the recruitment and activation of T cells, it concomitantly increased the expression of PD-1, which hindered the anticancer effects of cytotoxic T cells (figure 1C). Therefore, we sought to combine OVH virotherapy and anti-PD-1 immunotherapy to compensate for their respective weaknesses. The combination of an anti-PD-1 antibody and OVH eradicated not only virus-injected tumors but also distant tumors in the Hepa1-6 tumor model when the tumors reached 250 mm^3^ (figure 1D–F). Mice treated with combination immunotherapy showed remarkably better overall survival than those treated with monotherapy, and all mice receiving the combination therapy survived without relapse during a 100-day follow-up period (figure 1G). The combination therapy more potently suppressed the growth of both treated tumors and distant untreated tumors than OVH or anti-PD-1 monotherapy. All mice became tumor-free after combination therapy administration, whereas no mouse became tumor-free after OVH or anti-PD-1 treatment. We also carried out depletion experiments to analyze the key immune cells involved in the combination therapy and discovered that CD8^+^ T cells played a predominant role in the response to the combination therapy and that macrophages and natural killer (NK) cells may be required for tumor regression (figure 1H–J). We used a tumor rechallenge model to investigate the effect of OVH treatment on the memory T cell response in vivo. Mice that were cured by OVH treatment proved to be fully immune to a secondary tumor rechallenge (all remained tumor-free and survived), which suggested that OVH treatment produced a long-term memory T cell response in vivo (figure 1K). Taken together, these results indicate that combination therapy incorporating OVH and anti-PD-1 therapy can overcome resistance to immunotherapy in immunosuppressive TME, resulting in enhanced anticancer effects.

### Construction and in vitro characterization of the OV YST-OVH

**Figure 2:**
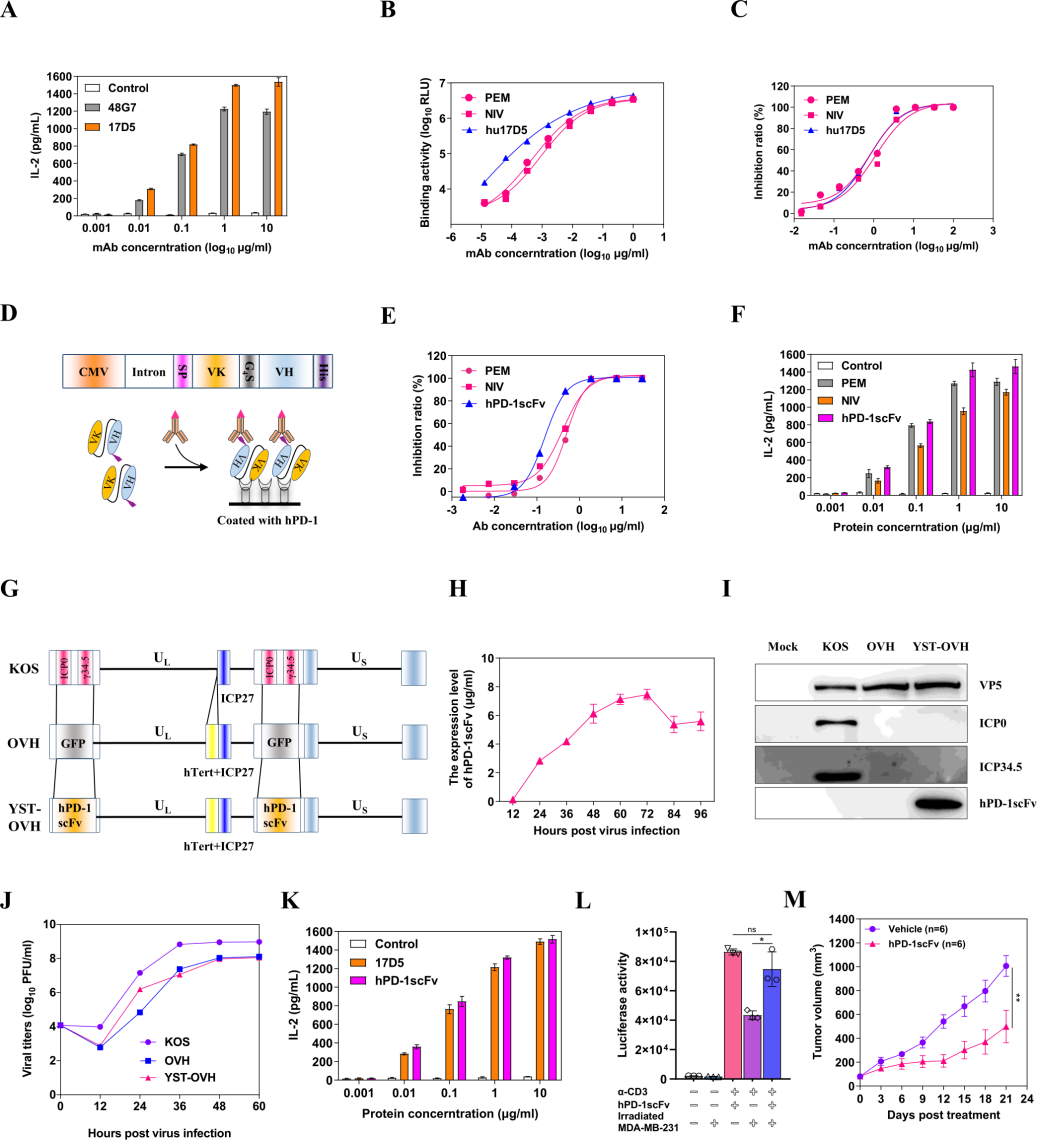
Generation of an oncolytic OVH virus expressing a single-chain variable fragment against PD-1. (A) A T cell activation assay was performed with serial dilutions of mouse anti-PD-1 antibodies. Activated human PBMCs were cocultured with 293T/hPD-L1 cells in the presence of increasing mouse anti-PD-1 antibodies for 3 days. The IL-2 levels in the supernatant were assayed by ELISA. (B) The reactivity of anti-PD-1 antibodies (PEM, NIV and hu17D5) against the human PD-1 protein was determined by an indirect CEIA. (C) The blocking activity of anti-PD-1 antibodies was determined by a blocking CEIA. (D) Genetic map showing the coding gene of the hPD-1scFv. The variable regions of the light chain and heavy chain coding genes of hu17D5 were linked by a G_4_S sequence, and expression was driven by the viral CMV promoter and a rabbit beta-globin intron. SP: a secretion signal sequence, VK: the variable region sequence of the light chain, VH: the variable region sequence of the heavy chain. (E) The blocking activity of hPD-1scFv and control anti-PD-1 antibodies was determined by a blocking CEIA. (F) The T cell activation activity of hPD-1scFv and control anti-PD-1 antibodies was determined by a T cell activation assay. (G) Schematic of the oncolytic viruses used in this study. Top: genetic map of wild-type HSV-1 (KOS strain). Middle: genetic map of OVH, with deletion of two copies of γ34.5 and ICP0, hTert promoter regulation of ICP27, and insertion of the GFP gene. Bottom: genetic map of YST-OVH showing the inserted coding gene of the hPD-1scFv. (H) hPD-1scFv yields from the supernatants of YST-OVH-infected U-2 OS cells at different time points (MOI=1). (I) Western blot analysis of various proteins in virus-infected cells. (J) Replication of the parental OVH strain and YST-OVH in U-2 OS cells compared with that of wild-type HSV-1. (K) The T cell activating activity of parental hu17D5 and purified hPD-1scFv from virus-infected cell supernatants was determined. (L) Jurkat-Lucia NFAT cells were cocultured with irradiated MDA-MB-231 cells and then stimulated with anti-CD3 and anti-CD28 antibodies in the presence of purified hPD-1scFv. Luciferase activity was measured 24 hours after stimulation. (M) The therapeutic efficacy of hPD-1scFv was evaluated in a syngeneic Hepa1-6 tumor model. Data represent results from one of two (M) independent experiments with n=6 per group. All values are presented as the mean±SEM; repeated-measure ANOVA (M) or unpaired two-tailed Student’s t-test (L). HSV-1, herpes simplex virus type 1. **p<0.01; CEIA, chemiluminescence immunoassay; OVH, oncolytic herpes simplex virus-1; YST-OVH, oncolytic herpes simplex virus-1 expressing PD-1 inhibitors; ANOVA, analysis of variance.

Since OVH was found to be an ideal companion to PD-1 blockade therapy and our previous proof-of-concept study demonstrated that a virus-delivered secreted PD-1 blockade agent might have the potential to treat cancers, it was our top priority to construct an OV armed with a humanized antibody that recognizes hPD-1 for further clinical investigation. To this end, we first developed a batch of monoclonal antibodies against hPD-1 (online supplemental figure S1A); among of them, one antibody (17D5) with high binding and blocking activities as well as T cell-activating activity was selected for further humanization development (online supplemental figure S1B, S1C and figure 2A). After complementarity determining region grafting and combinatorial library screening, we obtained a humanized monoclonal antibody, hu17D5, that had higher binding activity than the commercially available anti-PD-1 antibodies pembrolizumab (PEM) and nivolumab (NIV) and similar blocking activity (figure 2B,C). Considering the capacity of the HSV-1 genome to carry an exogenous gene and the expression efficiency of the encoding gene, we ultimately chose a single-chain antibody fragment over a full-length antibody as the gene for viral delivery. Based on the gene sequences of hu17D5, we then constructed an expression cassette expressing hPD-1scFv, with a His tag at the C-terminus (figure 2D). We examined the expression of hPD-1scFv in the supernatant and purified it for functional evaluation (online supplemental figure S2A). As expected, purified hPD-1scFv exhibited stronger blocking activity and T cell-activating activity than commercially available PD-1 antibodies, which suggested that this recombinant protein was a good candidate for therapeutic applications (figure 2E,F, online supplemental figure S2B and S2C). It was found that hPD-1scFv specifically reacted with PD-1 of human or nonhuman primate origins but not with PD-1 of mouse origin (online supplemental figure S2D).

10.1136/jitc-2022-004762.supp1 Supplementary data

Next, we constructed a novel OV carrying the hPD-1scFv gene in the backbone of the tumor-selective OV OVH. This new virus was named YST-OVH (figure 2G). We verified the expression of the therapeutic hPD-1scFv protein in the supernatant of YST-OVH-infected cells. The quantity of the hPD-1scFv protein was determined by ELISA, and the results showed that the expression of the hPD-1scFv protein peaked at 72 hours after infection (figure 2H). Then, we compared the replication efficiency of this virus with that of the parental virus in cancer cells. As shown in figure 2I, these two viruses exhibited almost identical replication kinetics. In addition, their oncolytic potency in U-2 OS cells and fusogenic plaque sizes were similar (online supplemental figure S3A, S3B). We compared the T-cell activating activity of parental hu17D5 and purified hPD-1scFv from virus-infected cell supernatants, and these two proteins showed almost identical activity (figure 2K). Moreover, hPD-1scFv restored the function of lymphocytes in a tumor-Jurkat cell co-culture (figure 2L), which demonstrated that blocking the PD-1/PD-L1 interaction with hPD-1scFv effectively abrogated the inhibition of immune cell functions by tumor cells.

Because of the approximately 61% amino acid identity shared by PD-1 of human and mouse origins, the protein we designed, hPD-1scFv, could not bind to mPD-1 and thus was not active in regular mice. Humanized PD-1 (PD-1-HU) transgenic mice have been developed by replacing the sequence encoding the mPD-1 extracellular domain (ECD) with that encoding the hPD-1 ECD and are a good in vivo model for validating the efficacy of anti-hPD-1 drugs. In this study, we used PD-1-HU mice to evaluate the antitumor immune response elicited by both the OV and hPD-1scFv. Finally, the antitumor efficacy of hPD-1scFv was validated in a Hepa1-6 tumor-bearing model established with PD-1-HU mice (figure 2M).

### Oncolytic effects and hPD-1scFv expression of YST-OVH in vitro and in vivo

**Figure 3:**
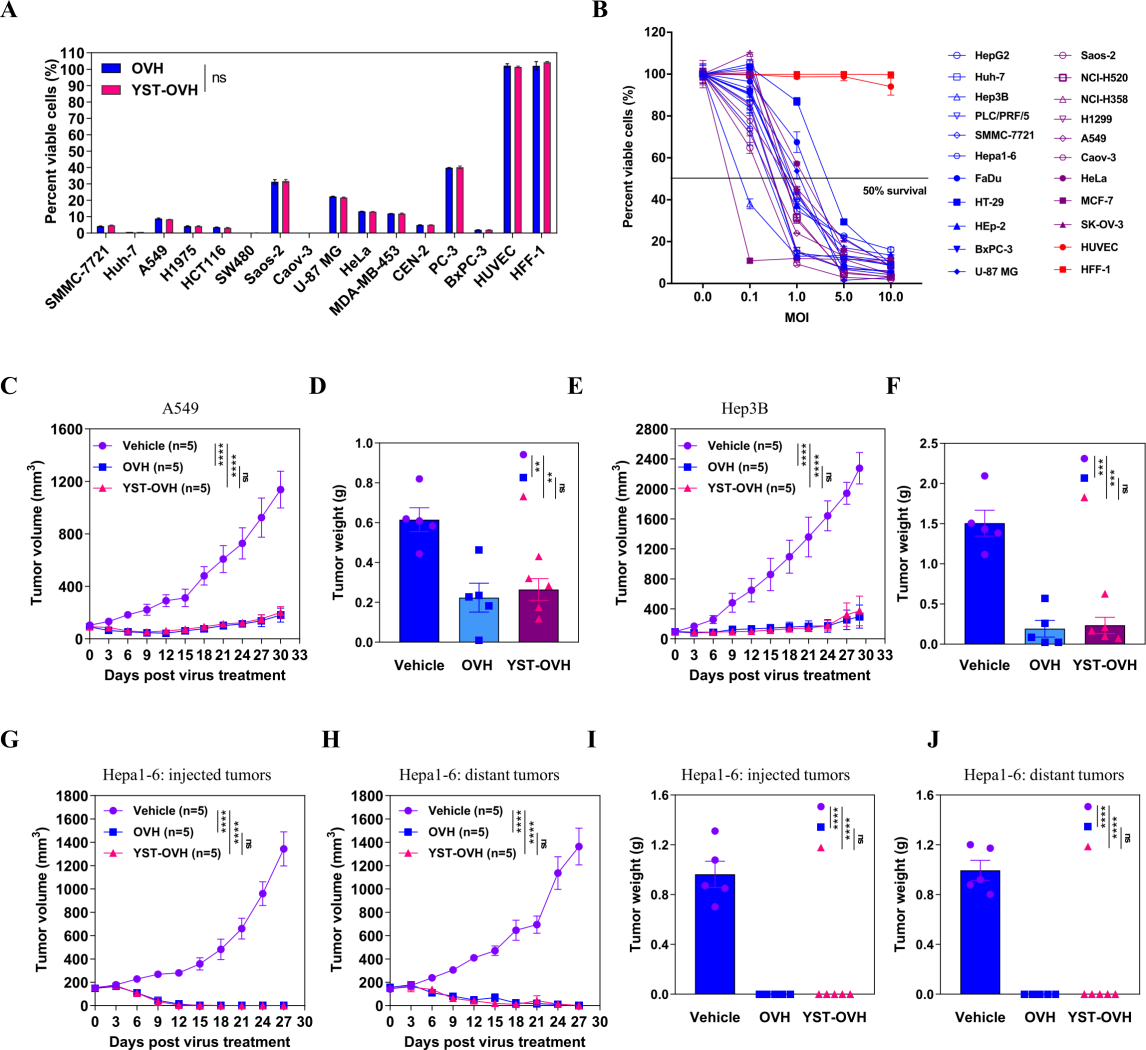
YST-OVH kills human and mouse cancer cells in vitro and in vivo. (A) Human cancer cells were infected with OVH or YST-OVH at an MOI of 1. Cell viability was analyzed at 3 days after infection by a CCK8 cell viability assay. (B) Human and murine cancer cells were infected with YST-OVH at the indicated MOIs. Cell viability was analyzed at 3 days after infection by a CCK8 cell viability assay. (C) Tumor growth of vehicle-treated, OVH-treated and YST-OVH-treated A549 tumors in immunodeficient nude mice. (D) Tumor weights of vehicle-treated, OVH-treated and YST-OVH-treated A549 tumors at 30 days post-treatment. (E) Tumor growth of vehicle-treated, OVH-treated and YST-OVH-treated Hep3B tumors in immunodeficient nude mice. (F) Tumor weights of vehicle-treated, OVH-treated and YST-OVH-treated A549 tumors at 30 days post-treatment. Bilateral flank Hepa1-6 tumor-bearing mice were received vehicle, OVH or YST-OVH therapy. The tumor growth of injected tumors (G) and distant tumors (H) is shown. The tumor weights of injected tumors (I) and distant tumors (J) at 27 days post-treatment are shown. Data either represent results from one of three (C–F) or one of two (G–K) independent experiments with n=5 per group. All values are presented as the mean±SEM; repeated-measure ANOVA (C, E, G, H) or unpaired two-tailed Student’s t-test (D, F, I. (J). ns, no significant differences; **p<0.01; ***p<0.001; ****p<0.0001. OVH, oncolytic herpes simplex virus-1; YST-OVH, oncolytic herpes simplex virus-1 expressing PD-1 inhibitors; ANOVA, analysis of variance.

To compare the oncolytic effects induced by YST-OVH and OVH, we first examined their tumor cell-killing effects on various cultured human cancer cell lines and normal cell lines. These two viruses exhibited almost identical oncolytic cytotoxicity (figure 3A). Of the 14 cancer cell lines we tested, at 72 hours after virus infection at a multiplicity of infection (MOI) of 1, 9 cell lines showed a more than 90% decrease in cell viability, and 5 cell lines showed a more than 70% decrease in cell viability. The two viruses both showed significantly reduced cytotoxicity to normal human cell lines. To determine whether the tumor cell-killing capability of YST-OVH depends on the infectious doses, we tested the cytotoxicity of YST-OVH at various infectious doses in different cell line sets. YST-OVH showed a great tumor cell-killing ability and tumor selectivity, and a positive correlation between the infectious doses and tumor cell-killing capability was observed (figure 3B). We also observed that hPD-1scFv expression in the supernatants of YST-OVH-infected tumor cells showed a dose-dependent increase (online supplemental figure S4A), which suggested that YST-OVH replicated well and produced gene products efficiently in vitro.

To further assess the in vivo tumor cell-killing potential of YST-OVH, subcutaneous (s.c.) xenograft mouse models established in nude mice and immunocompetent C57BL/6 mice were used for preliminary evaluation, although hPD-1scFv expressed from YST-OVH was not active in these models. The in vivo oncolytic effects of YST-OVH and OVH were first evaluated in subcutaneous xenograft model mice implanted with human cancer cell lines (Hep3B and A549). After three intratumoral (i.t.) injections of virus or vehicle, comparable tumor inhibition was observed in the OVH-treated mice and YST-OVH-treated mice (figure 3C–F). The oncolytic effects of YST-OVH on an HCT116 mouse model were highly dependent on OV dose (online supplemental figure S4B), and hPD-1scFv expression in the serum of YST-OVH-treated mice showed a dose-dependent increase, which demonstrates that oncolysis can cause hPD-1scFv produced in tumor cells to be released into the serum (online supplemental figure S4C). We then established bilateral flank tumor models implanted with mouse Hepa1-6 cancer cells. Viral administration to the right flank tumor (200 mm^3^) resulted in complete tumor regression in both flanks, and no obvious efficacy difference was observed between the OVH-treated mice and YST-OVH-treated mice (figure 3G–J), which was possibly due to the relatively small sized tumors. Moreover, we also verified that the expression of the hPD-1scFv protein in the serum and tumors of YST-OVH-treated mice varied in a dose-dependent manner (online supplemental figure S5A, S5B). Taken together, these results indicate that YST-OVH replicates as well as OVH in multiple types of tumors and the viruses show similar oncolytic effects.

### YST-OVH improves systemic tumor control and enhances effector T cell function in PD-1-HU mice

To evaluate the systemic efficacy of delivering the antibody hPD-1scFv to the TME with YST-OVH in immunocompetent and humanized PD-1 mouse models, we established an orthotopic tumor model and a bilateral flank tumor model in immunocompetent PD-1-HU mice.

**Figure 4:**
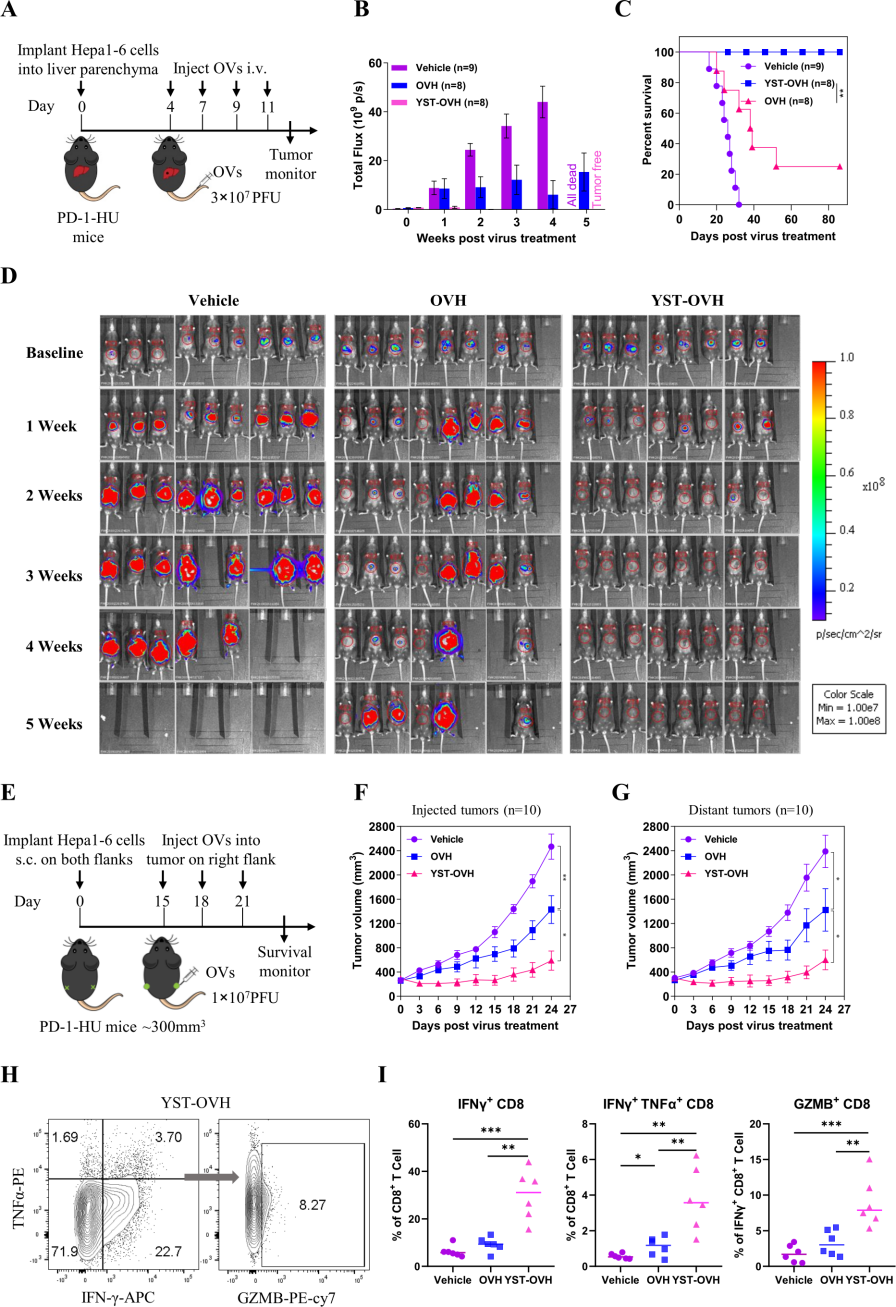
YST-OVH potentiates antitumor efficacy and enhances CD8^+^ T cell activation. (A) Treatment scheme for an orthotopic tumor model. An orthotopic tumor model was established by intrahepatic injection of mouse Hepa1-6-luc cells (expressing luciferase) into immunocompetent PD-1-HU mice. Four days later, the mice were intravenous injected with a saline vehicle control or 3×10^7^ PFU of OVH or YST-OVH. The indicated treatments were taken 4, 7, 9 and 11 days post-tumor implantation. (B) Luciferase fluorescent signals were recorded 1, 2, 3, 4 and 5 weeks after the indicated treatments. (C) Survival of Hepa1-6-luc tumor-bearing mice. (D) Luciferase imaging of mice was performed 1, 2, 3, 4 and 5 weeks after the indicated treatments. (E) Treatment scheme for bilateral flank Hepa1-6 tumor-bearing PD-1-HU mice. Mice bearing tumors received vehicle, OVH or YST-OVH therapy. The tumor growth of injected tumors (F) and distant tumors (G) are shown. (H) Mice bearing Hepa1-6 tumors were intratumorally injected with vehicle, OVH or YST-OVH, and the tumors were collected on day 16 after virus injection and analyzed by flow cytometry. (I) The percentages of tumor-infiltrating IFN-γ^+^, IFN-γ^+^TNF-α^+^, and GZMB^+^CD8^+^ T cells in the tumor CD8^+^ T cell population. Data represent results from one of three (A, E, H) independent experiments with n=6 to n=10 per group. Data for survival were analyzed by the log-rank (Mantel-Cox) test (C). All values are presented as the mean±SEM; repeated-measure ANOVA (F, G) or unpaired two-tailed Student’s t-test (I). *p<0.05; **p<0.01; ***p<0.001. OVH, oncolytic herpes simplex virus-1; YST-OVH, oncolytic herpes simplex virus-1 expressing PD-1 inhibitors; ANOVA, analysis of variance; PFU, plaque-forming units.

By intrahepatic injection of firefly luciferase gene-expressing Hepa1-6 cells into PD-1-HU mice, we established an orthotopic tumor model and to compare the effectiveness of intravenous administration of YST-OVH against intravenous administration of OVH. Beginning 4 days after tumor implantation, the mice received four intravenous injections of virus or vehicle. Tumor progression was monitored by luciferase-based imaging starting on day 7 post tumor implantation (figure 4A). Treatment of these mice with YST-OVH significantly inhibited tumor growth and increased the overall survival time compared with mouse treatment with OVH or the vehicle control (figure 4B–D). All mice receiving YST-OVH therapy survived without relapse during an 86-day follow-up period (figure 4B,C).

By s.c. injection of Hepa1-6 cells into PD-1-HU mice, we established a bilateral flank tumor model to compare the effectiveness of i.t. administration of YST-OVH against i.t. administration of OVH (figure 4E). Once the Hepa1-6 tumors reached 350 mm^3^, the xenografted mice were randomized to receive i.t. injection of three doses of virus or vehicle in the right flank. YST-OVH treatment induced more robust tumor regression than OVH treatment and resulted in better tumor rejection not only in the virus-injected tumors but also in the distant flank tumors (figure 4F,G). We also used a less immunogenic 4T1 tumor model to test the effectiveness of YST-OVH (online supplemental figure S6A, S6B).^26 27^ Although YST-OVH replicated worse in 4T1 cells than Hepa 1–6 cells, YST-OVH treatment also induced more robust tumor regression than OVH treatment in the 4T1 s.c. tumor model (online supplemental figure S6C, S6D).

We evaluated the infiltration of immune cells after i.t. injection of the OVs in the Hepa1-6 subcutaneous tumor model. Flow cytometric analysis showed that i.t. injection of YST-OVH significantly induced the expansion of activated TILs, including IFN-γ^+^CD8^+^ T cells, IFN-γ^+^TNF-α^+^CD8^+^ T cells and GZMB^+^CD8^+^ T cells, compared with either OVH or vehicle treatment (online supplemental figure S7, figure 4H,J). Consistent with previous results showing that the administration of anti-PD-1 antibodies can rejuvenate tumor-infiltrating CD8^+^ T cells, our results indicated that YST-OVH could enhance antitumor efficacy by activating tumor-infiltrating CD8^+^ T cells.

### YST-OVH modifies the immune landscape of tumors

**Figure 5:**
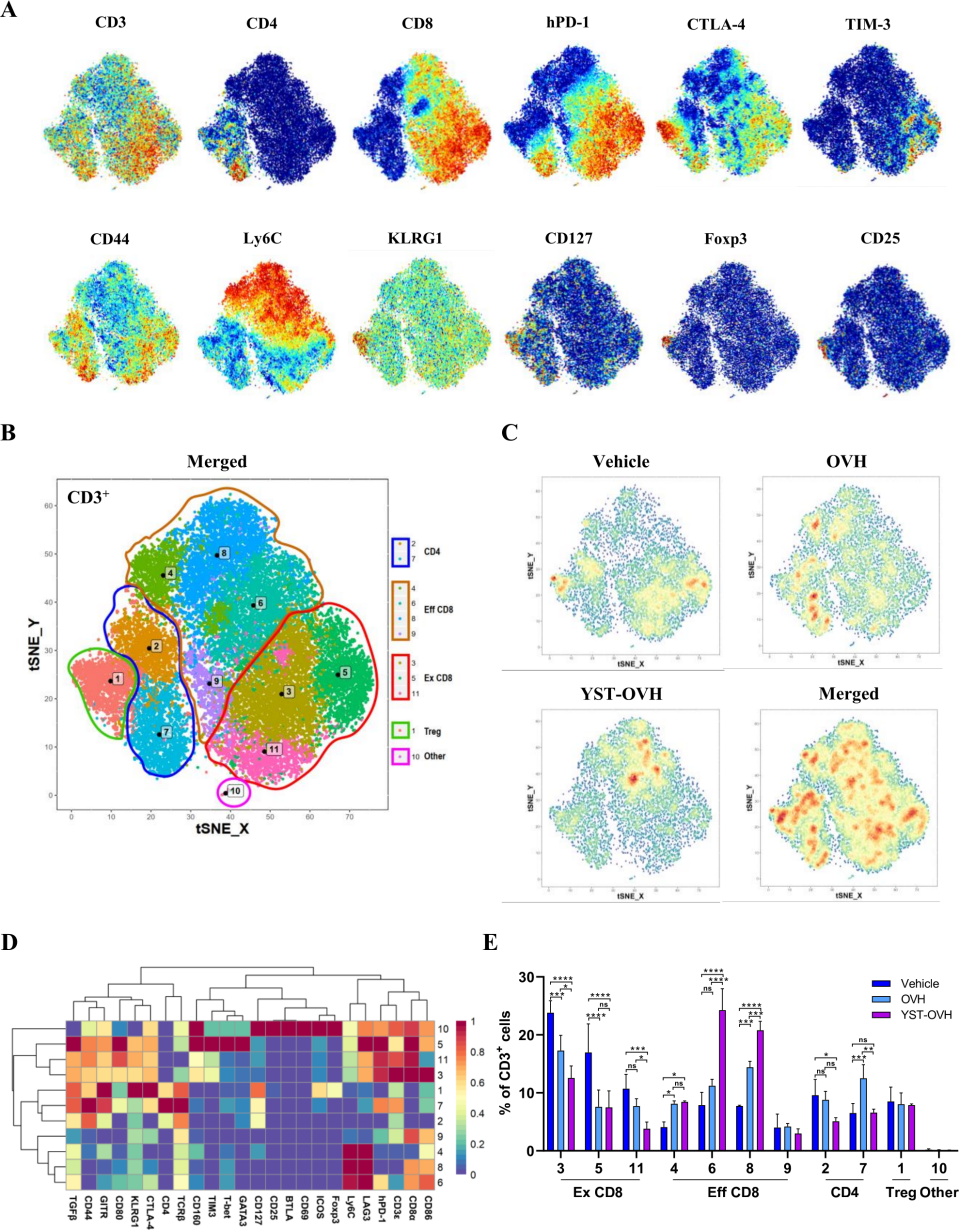
YST-OVH modifies the immune landscape of tumors and reinvigorates the antitumor T cell response. (A) t-SNE plot of CD3^+^ Hepa1-6 tumor-infiltrating T cells overlaid with the expression of selected markers. (B) t-SNE plot derived from CyTOF analysis of tumor-infiltrating T cells obtained from each treatment group. Cells are colored by the clusters identified by PhenoGraph. (C) Density t-SNE plots of an equal numbers of tumor-infiltrating T cells from each treatment group. (D) Heatmap displaying the normalized marker expression of each T cell cluster. (E) Quantitative analysis of the T cell clusters as a percentage frequency of CD3^+^ T cells. Quantitative data are presented as the mean ± SEM and were analyzed by an unpaired two-tailed Student’s t-test. CyTOF, cytometry by time-of-flight. ns, no significant differences; *p<0.05; **p<0.01; ***p<0.001; ****p<0.0001. OVH, oncolytic herpes simplex virus-1; YST-OVH, oncolytic herpes simplex virus-1 expressing PD-1 inhibitors; t-SNE, t-Distributed Stochastic Neighbor Embedding.

We next sought to determine how immunity contributes to the superior tumor control capability of YST-OVH, and we used CyTOF to dissect the precise dynamics in the TME. To generate a comprehensive view of the TIL populations, we designed a staining panel with 32 surface and 6 intracellular markers. This panel included non-T cell lineage markers (eg, CD11b, CD11c, CD19 and NK1.1), T cell differentiation markers (eg, CD44, CD62L, Ly6C and FOXP3), and T cell activation and inhibition markers (eg, PD-1, TIM-3, CTLA-4 and LAG-3). PhenoGraph analysis of the expression profiles of the 38 cell markers identified 15 main immune cell subsets: CD4^+^ T cells (T1), CD8^+^ T cells (T4 and T8), B cells (T7), NK cells (T3), myelocytic myeloid-derived suppressor cells (M-MDSCs) (T5 and T11), granulocytic myeloid-derived suppressor cells (PMN-MDSCs) (T6), macrophages (T12, T14 and T15), dendritic cells (T9 and T13), and other CD3^+^ cells (T2 and T10). As shown in online supplemental figure S8A-E, OV treatment had significant effects on the proportions of CD8^+^ T cells, macrophages and PMN-MDSCs within the entire population of CD45^+^ cells. Interestingly, treatment with OVH but not YST-OVH was associated with a significant increase in the PMN-MDSC subset. PMN-MDSCs have immunosuppressive activity and restrict immune responses to cancer immunotherapy. These results indicated that YST-OVH treatment did not recruit a large number of immunosuppressive cells into the TME compared with OVH. Moreover, our previous study and available publications both indicated that HSV-1 based virotherapy and PD-1 blockade therapies relied primarily on T cells for their antitumor effects.^10 22^ Thus, we decided to focus our analysis on the T cell compartment. When individual markers were highlighted in the t-SNE plots, the key markers for T cell activation, differentiation, and exhaustion were colocalized (figure 5A). PhenoGraph analysis identified 10 main T cell subsets: CD8^+^ T cells (T3, T4, T5, T6, T8, T9 and T11), CD4^+^ T cells (T2 and T7), Treg cells (T1), and other CD3^+^ cells (T10) (figure 5B). Analysis of those T cell clusters revealed dramatic population shifts in response to OVH or YST-OVH therapy (figure 5C). The expression profiles of 24 different markers on each T cell cluster were visualized in a heatmap (figure 5D). Figure 5E shows the proportions of each T cell cluster among the entire tumor-infiltrating T cell population. Notably, both OVH treatment and YST-OVH treatment led to a significant shift in T cell populations, with YST-OVH significantly increasing the T6 and T8 proportions. Three phenotypically exhausted PD-1^hi^CTLA-4^+^TIM-3^+^ populations (T3, T5 and T11) were the most expanded among the CD8^+^ populations in the vehicle-treated group. OVH treatment significantly reduced the proportions of the exhausted CD8^+^ T cell populations (Ex CD8; T3, T5 and T11). Surprisingly, the magnitude of this decrease was greater following YST-OVH treatment (T3 and T11). We next assessed the effect of OV treatment on CD44^+^CD62L^lo^ effector CD8^+^ T cell populations (Eff CD8). Contrary to the changes in Ex CD8 populations, OV treatment significantly increased the proportions of the Eff CD8 T cell populations (T4, T6, and T8). Moreover, the proportion of Eff CD8 was much higher in the YST-OVH-treated group than in the OVH-treated group (T6 and T8). Interestingly, the Eff CD8 elicited by YST-OVH treatment had high-expression of Ly6C, a marker of memory CD8^+^ T cells.^28^ We next assessed if intratumoral OVs induced tumor-specific and virus-specific CD8^+^ T cells in tumors. Tumor-specific and HSV-1-specific memory CD8^+^ T cells in tumors were quantified by direct quantification of tumor-specific memory CD8^+^ T cells in harvested tumors using OVA-specific and HSV-1-specific multimers. Interestingly, YST-OVH treatment can induce strong anti-tumor memory CD8^+^ T cells but very weak anti-viral memory CD8^+^ T cells on day 9 post-treatment (online supplemental figure S8F, S8G). These results indicated that YST-OVH treatment predominantly affected CD8^+^ T cells compared with OVH or vehicle treatment, and might reshape the TME by reprogramming Eff CD8 into memory T cells.

YST-OVH reversed CD8^+^ T cell exhaustion and increased the proportion of memory CD8^+^ T cells, which is extremely beneficial for inducing improved antitumor immunity. Moreover, all Ex CD8 clusters, which were decreased by YST-OVH treatment, had high coexpression of CTLA-4 and TIM-3, which are common inhibitory receptors that suppress T cell function. T cell exhaustion is often characterized by high expression of immune inhibitory receptors and loss of T cell function, which could further reduce the antitumor activity of CD8^+^ T cells.^29^ Unexpectedly, neither OVH or YST-OVH had a significant effect on the proportion of KLRG1^+^CTLA-4^+^ Treg cells (T7). These activated Treg cells could dampen the YST-OVH induced antitumor immune response. These results suggest that reversing T cell exhaustion and blocking activated Treg cells by appropriate immune checkpoint blockade (eg, anti-CTLA-4 or anti-TIM-3 therapy) may further promote the antitumor effect of YST-OVH treatment.

### YST-OVH treatment increases tumor immunogenicity and systemic sensitivity to immune checkpoint blockade

**Figure 6:**
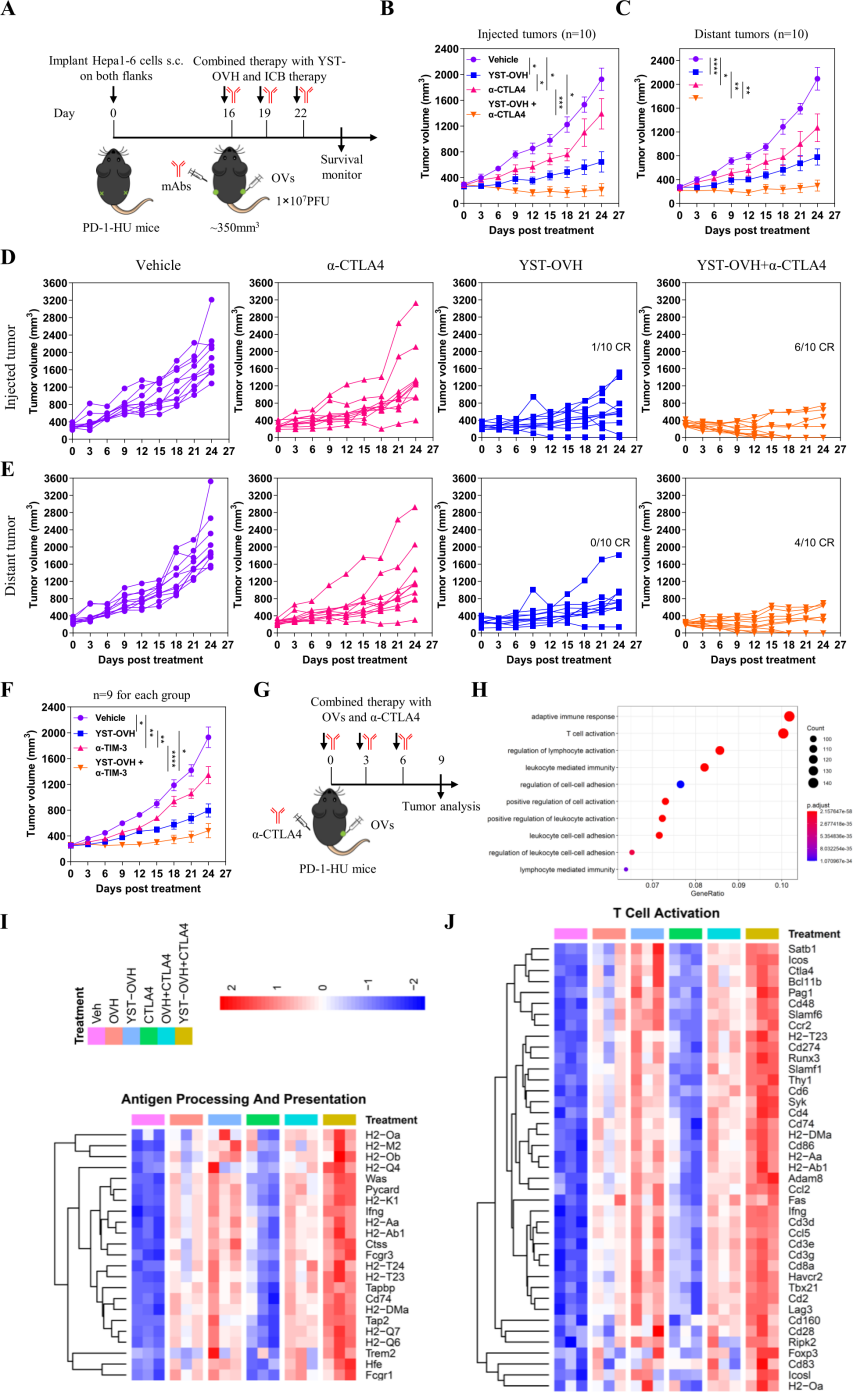
Therapeutic delivery of PD-1 inhibitors by YST-OVH potentiates the antitumor efficacy of immune checkpoint blockade. (A) Treatment scheme for bilateral flank Hepa1-6 tumor-bearing PD-1-HU mice. Mice bearing tumors received monotherapy or combination therapy with YST-OVH and anti-CTLA-4. Tumor growth of injected tumors (B) and distant tumors (C). (D) Individual tumor growth curves of injected tumors. (E) Individual tumor growth curves of distant tumors. (F) Treatment scheme for a syngeneic Hepa1-6 tumor model. Mice-bearing tumors received monotherapy or combination therapy with YST-OVH and anti-TIM-3. Tumor growth was monitored over a 27-day period. (G) Treatment scheme for a syngeneic Hepa1-6 tumor model. Mice-bearing tumors received monotherapy or combination therapy with OVs and anti-CTLA-4, and tumors were collected on day 9 post-treatment and subjected to RNA-seq analysis. (H) GO enrichment analysis showing the top 10 most enriched pathways. (I) Heatmap showing the expression of differentially expressed antigen processing and presentation genes. (J) Heatmap showing the expression of differentially expressed T cell activation genes. Gene expression was normalized to values obtained for an untreated control. Data represent results from one of three (A) or one of two (F) independent experiments with n=9 to n=10 per group. All values are presented as the mean±SEM; repeated-measure ANOVA (B, C, F). CR, complete response; OVs, oncolytic viruses; PR, partial response. *p<0.05; **p<0.01; ***p<0.001; ****p<0.0001. YST-OVH, oncolytic herpes simplex virus-1 expressing PD-1 inhibitors; ANOVA, analysis of variance; PFU, plaque-forming units.

To explore whether combination with anti-CTLA-4 could enhance the antitumor efficacy of YST-OVH, we performed i.t. injection of YST-OVH with intraperitoneal (i.p.) injection of anti-CTLA-4 to treat the Hepa1-6 bilateral flank tumor model (figure 6A). As shown in figure 6B,C, monotherapy with YST-OVH or anti-CTLA-4 significantly inhibited the tumor growth in both flanks. Although there was no significant difference in the distant tumor volume between the two monotherapies (figure 6C), YST-OVH therapy showed a trend toward a smaller virus-injected tumor volume than anti-CTLA-4 therapy (figure 6B), possibly due to the direct oncolytic effects of YST-OVH in the virus-injected tumors. However, combination therapy with YST-OVH and anti-CTLA-4 further reduced the volume of the tumors in both flanks, and four mice achieved a complete response (CR) (figure 6D,E). Six-tenths of the cotreated mice achieved a CR for the virus-injected tumor, and 4/10 of the cotreated mice achieved a CR for the distant tumors, while only 1/10 of YST-OVH-treated mice achieved a CR for the virus-injected tumors, and none of the mice treated with anti-CTLA-4 therapy or vehicle achieved a CR or partial response (PR). Next, we evaluated the antitumor efficacy of the combined application of YST-OVH and anti-TIM-3. Monotherapy with YST-OVH or anti-TIM-3 significantly inhibited tumor growth, and YST-OVH therapy showed a trend toward a smaller tumor volume than anti-TIM-3 therapy (figure 6F). Combination therapy with YST-OVH and anti-TIM-3 more potently suppressed tumor growth than YST-OVH or anti-TIM-3 monotherapy. These results indicated that rational combination treatment with anti-CTLA-4 or anti-TIM-3 further improved the antitumor efficacy of YST-OVH.

To investigate the underlying mechanisms by which YST-OVH potentiates other checkpoint blockade therapies, we dissected the gene alterations in the tumors of mice receiving YST-OVH and/or anti-CTLA-4 therapy compared with those mice in the tumors of mice treated with OVH and/or anti-CTLA-4 therapy (figure 6G). Gene ontology (GO) analysis of RNA-sequencing (RNA seq) data revealed that several immune-related signaling pathways, such as adaptive immune response, T cell activation, lymphocyte activation and leucocyte-mediated immunity, were significantly enriched in the cotreated tumors (figure 6H). Consistent with the previous results, these data suggested that combination therapy with YST-OVH and anti-CTLA-4 promoted T cell activation and induced a more potent antitumor response. We also observed that genes involved in antigen processing and presentation, T cell activation and cell killing were significantly upregulated in the YST-OVH and anti-CTLA-4 cotreated tumors compared with tumors treated with either monotherapy or OVH and anti-CTLA-4 cotreatment (figure 6I, online supplemental figure S9A), while genes involved in the cell cycle checkpoint were significantly downregulated (figure 6I, online supplemental figure S9B). YST-OVH therapy could potentiate anti-CTLA-4 therapy by promoting tumor antigen processing and presentation and T cell activation more potently than OVH therapy. These results indicate that the combined application of YST-OVH and anti-CTLA-4 may further improve antitumor efficacy by increasing tumor immunogenicity.

### YST-OVH shows an outstanding safety profile in nonhuman primates

To determine the safety profile of YST-OVH and further translate YST-OVH into the clinic, we evaluated the neurovirulence of intrathalamic (i.c.) injection of YST-OVH in rhesus macaques and the systemic toxicity of intravenous injection of YST-OVH in cynomolgus monkeys.

In the study evaluating the neurovirulence of YST-OVH, five males (Rhe1-5) and five females (Rhe6-10) were included in the treatment group and two animals of different sexes (CTL1-2) were included in the control group (online supplemental table S1). Animals were challenged with one left i.c. injection and one right i.c. injection of the virus at a dose of 1.65×10^7^ PFU/injection (online supplemental figure S10A). The clinical symptoms, weight and temperature of the treated animals were monitored over a 22-day period. The results showed slight diarrhea in two YST-OVH-treated animals (Rhe2 and Rhe6), but no other clinical symptoms occurred (online supplemental table S2). No individuals in the treatment group had any abnormal changes in body weight or temperature during the experiment (online supplemental figure S10B, S10C).

In the study evaluating the toxicity of YST-OVH, five males (C1-5) and five females (C6-10) were included in the treatment group, and five males (Y1-5) and five females (Y6-10) were included in the control group (online supplemental table S3). Animals were challenged with three sequential treatment cycles of intravenous infusion of YST-OVH (2.4×10^8^ PFU per dose). Each treatment cycle contained five injections, and the injections were performed every other day (online supplemental figure S11A). The clinical symptoms, weight and temperature of YST-OVH-treated animals were monitored over a 58-day period. Half of the animals were euthanized on day 30 and then evaluated by histological examinations. The results showed slight diarrhea in one YST-OVH-treated animal (Y1) and one vehicle-treated animal (C5), and ruffled fur was observed in two YST-OVH-treated animals (Y6 and Y8) and one vehicle-treated animal (C5), but no other clinical symptoms occurred (online supplemental table S4). No individuals in the treatment group had any abnormal changes in body weight or temperature during the experiment (online supplemental figure S11B, S11C). Serum biochemistry studies demonstrated no significant difference in the total bilirubin (T-Bil), creatinine (CRE), creatine kinase (CK), blood urea nitrogen (UREA), or albumin (Alb) levels between the YST-OVH-treated group and the vehicle-treated group during the course of the study (online supplemental figure S11D). The results showed that alanine aminotransferase and aspartate aminotransferase activities were slightly elevated in one YST-OVH-treated animal (Y4) after the first cycle of intravenous injection of YST-OVH and another YST-OVH-treated animal (Y5) after the final intravenous injection of YST-OVH, but they returned to normal after several days. Hematological studies demonstrated no overt leukocytopenia, leucocytosis or anemia and no abnormalities in the platelet and neutrophil counts (online supplemental figure S11E). We observed that several test parameters slightly changed during the course of the experiments, but they fluctuated within the normal range. We also observed no significant difference in cytokine expression between the YST-OVH-treated group and the vehicle-treated group during the course of the study (online supplemental figure S11F). On day 30, histological analysis of vital tissues from the YST-OVH-treated group and vehicle-treated group, including the brain, heart, lungs, liver, sternum marrow, and kidneys, was performed by H&E staining. No obvious pathological abnormalities were observed (online supplemental figure S12). All the results support the conclusion that YST-OVH exhibits an excellent safety profile and is well-tolerated in nonhuman primates, thus providing important safety evidence for further clinical translation of YST-OVH virotherapy.

### YST-OVH therapy shows superior antitumor activity in a humanized mouse model

**Figure 7:**
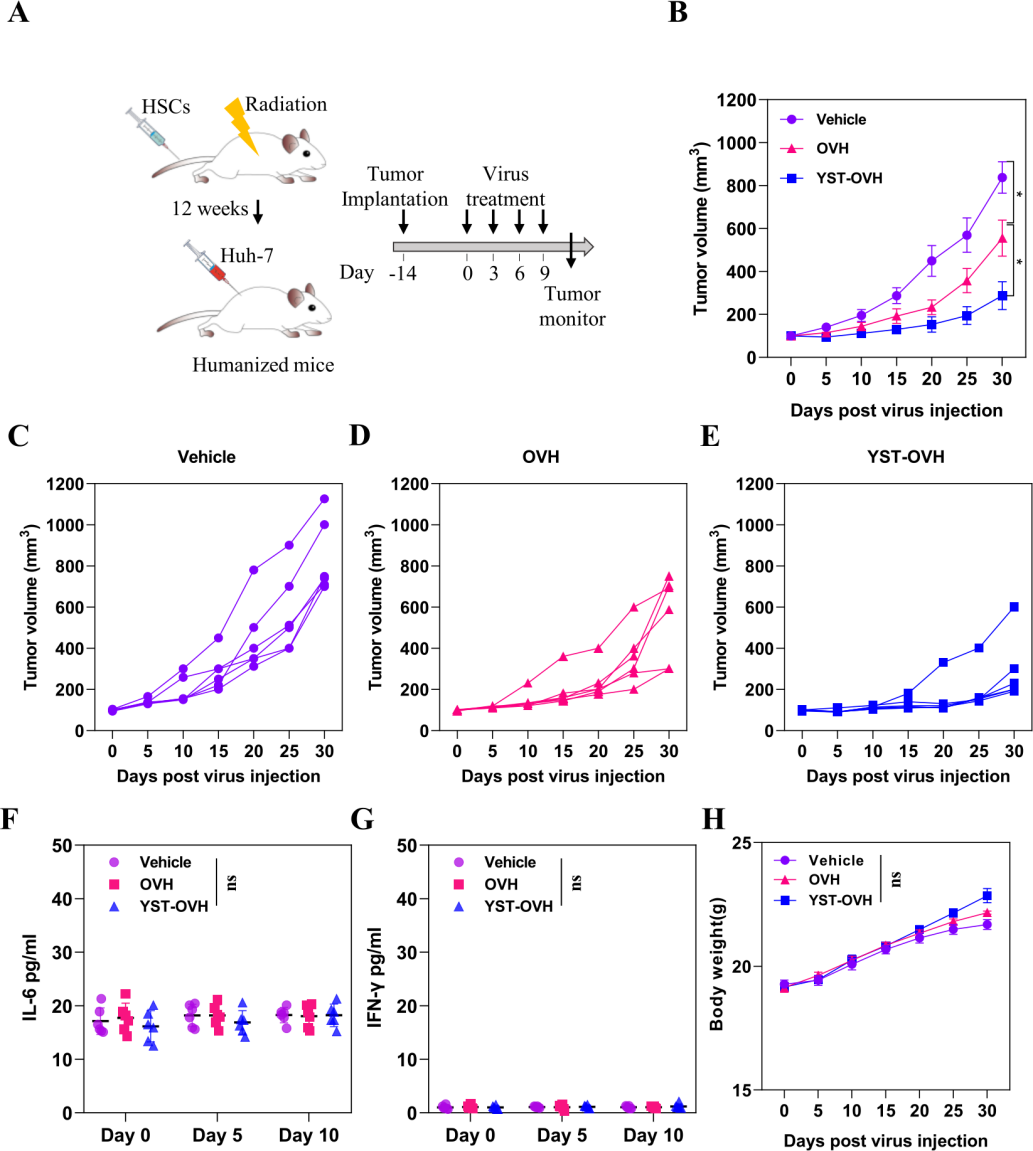
YST-OVH treatment inhibits tumor progression in humanized mice without induction of strong inflammatory responses, such as CRS. (A) Treatment scheme for a humanized mouse model. (B) Mice bearing Huh-7 tumors were intratumorally injected with vehicle, OVH or YST-OVH, and tumor growth was monitored over a 30-day period. Individual tumor growth curves of vehicle injected (C), OVH injected (D) and YST-OVH injected (E) tumors. Blood was collected at 0, 5 and 10 days after treatment for plasma cytokine analysis. (F) The IL-6 level was determined by ELISA assay. (G) The IFN-γ level was determined by ELISA assay. (H) The body weights of the experimental mice were recorded every 5 days. All values are presented as the mean±SEM; repeated-measure ANOVA (B), one-way ANOVA (F–H). ns, no significant differences; *p<0.05; OVH, oncolytic herpes simplex virus-1; YST-OVH, oncolytic herpes simplex virus-1 expressing PD-1 inhibitors; ANOVA, analysis of variance.

We finally tested the effectiveness of YST-OVH using a humanized mouse model. Huh-7 human liver cancer cells were implanted subcutaneously into an immunodeficient genetically humanized mouse model NOD-*Prkdc* ^scid^ *Il2rg* ^em1^/Smoc (NSG) mice, which are optimized for the engraftment and function of introduced human hematopoietic stem cells (HSCs) (figure 7A). To generate NSG mice with functional human T cells, human CD34^+^ HSCs were implanted subcutaneously into these mice.^30–32^ The successful generation of human immune cell populations in the NSG mice was validated by flow cytometry, and humanized mice with over 25% human CD45^+^ cell reconstitution were used for the tumor study (online supplemental figure S13). Before tumor implantation, the mice were randomized into treatment groups based on HSC donors and human immune cell engraftment. YST-OVH was significantly more effective than OVH at inhibiting the progression of liver tumors in vivo (figure 7B–E).

Consistent with the above studies in cynomolgus monkeys, no cytokine elevations or body weight changes were observed in tumor-bearing humanized mice treated with YST-OVH or OVH (figure 7F–H). The i.t. expression of hPD-1 scFv by YST-OVH did not significantly increase the IFN-γ and IL-6 levels (p>0.05) detected in the serum on days 5 and 10 after treatment, showing no toxicity associated with cytokine release syndrome (CRS). Overall, these results suggest that YST-OVH markedly enhances antitumor efficacy and is likely to be well tolerated in humanized mouse models.

## Discussion

Cancer immunotherapy with the immune checkpoint blockade agents, such as PD-1/PD-L1 antibodies, has been widely used in the clinic against certain kinds of cancers.^6^ However, the effectiveness of these drugs in other cancers is still unsatisfactory. It has been clarified that many factors operating within the TME contribute to these limited outcomes; these factors include the recruitment of immunosuppressive immune cells (including tumor-associated macrophages (TAMs), MDSCs, and Treg cells), a lack of neoantigens and low T cell infiltration into the tumor.^23^ These factors work together to suppress the effects of immune checkpoint blockade. Accumulating evidence now shows that the therapeutic efficacy is greatly improved by the combination of oncolytic virotherapy enhancing T cell infiltration and reducing immunosuppressive immune cells.^33^ OV treatment is the key factor in this regimen, and OVs have several advantages related to inducing extensive effects on the TME of solid tumors, especially noninflamed tumors, including direct tumor cell killing, recruitment of the innate and adaptive cells, and activation of the T cells.^34^ Furthermore, OVs reverse the immune balance within T cells and lead to an increased ratio of effector T cells to Treg cells.^12^

However, OV treatment could alter the cancer-immune set point in tumors to attenuate the increased immune response and thus allow resumption of rapid tumor growth at a later stage, leading to limited therapeutic outcomes; this suggests the necessity of developing novel strategies to maximize the potential of oncolytic virotherapy.^35^ Recently, the development of OVs as engineered oncolytic vectors has accelerated. Accumulating evidence has established a viable strategy for the rational design of ‘arming combination therapy’ by targeting factors in the TME, including ICOS, IL-12 and E-cadherin.^18 21 36^ Signaling through PD-1 inhibits T cell activation, and was shown to be critical for T cell-dependent antitumor responses induced by oncolytic virotherapy. These studies, however, indicated the possibility that targeting additional signaling through a deregulated PD-1 target in the TME could further enhance the therapeutic efficacy of OVH virotherapy. The activity of immunomodulators expressed by OVs may be critical for achieving synergistic antitumor effects. In a preclinical glioblastoma study, the use of HSV-1 viruses expressing an scFv against mPD-1 produced slightly improved mouse survival.^37^ Our previous study showed that a recombinant HSV-1 virus expressing an scFv against mPD-1 could locally deliver an aMPD-1 scFv in the TME to achieve significantly improved mouse survival and reduced tumor burdens. However, these studies served as surrogates for evaluating OVs for clinical investigation and lacked careful evaluations in clinically relevant models. In this study, we developed a functional hPD-1 blocker, hPD-1scFv, that was at least noninferior to commercially available anti-PD1 antibodies and should have the potential to immunomodulate intratumoral T cells. Indeed, when tested in vivo, recombinant HSV-1 expressing hPD-1scFv demonstrated therapeutic superiority over the parental OVH virus, with both producing an enhanced local effect in injected tumors and an abscopal effect in distant tumors. These effects were associated with increases in the expansion and activation of CD8^+^ T cells. The most pronounced differences, however, were seen in the absolute numbers of IFN-γ^+^CD8^+^ T cells, IFN-γ^+^TNF-α^+^ CD8^+^ T cells and GZMB^+^CD8^+^ T cells. Our preclinical results also showed that YST-OVH therapy was not only effective in relatively high immunogenic Hepa 1–6 tumors but also in less immunogenic (cold) 4T1 tumors, suggested that YST-OVH therapy maybe a promising drug candidate for treating poorly immunogenic tumors.

As expected, our data from the multiple animal models clearly reflected these synergistic effects, demonstrating the effectiveness of this novel virotherapy as a standalone treatment compared with the possible dual combinations, which were themselves better than the corresponding single-agent treatments. The powerful antitumor immune responses were tumor-antigen specific and durable, as all survivors completely rejected a second tumor when rechallenged and survived without any tumor development. The immune basis of this therapy was illustrated by the following two mechanisms: (1) early modifications to the immunosuppressive TME and the establishment of a systemic response were achieved with OVs, and (2) the exhausted microenvironment of responder mice was further inflamed by rescue of T cells with the virus expressing hPD-1scFv. Notably, OVs armed with PD-1 blockers but not unarmed OVs could elicit effective antitumor activity in the late-stage tumor model (tumor volume >350 mm^3^), which suggests that only an appropriately armed OV can effectively modulate this more immunosuppressive TME, as supported by other recent studies.^38^ Here, we also demonstrated that intratumoral expression of hPD-1scFv by YST-OVH favored antitumor responses via not only inhibition of the PD-1/PD-L1 axis in the TME but also alteration of the cancer-immune set point. Notably, YST-OVH treatment upregulated CTLA-4 and TIM-3 expression on exhausted CD8^+^ T cells and resulted in high levels of CTLA-4^+^ Treg cells, and these changes were likely induced by intratumoral expression of hPD-1scFv. Although CTLA-4 and TIM-3 generally downregulate T cell activity, our data demonstrated that YST-OVH combined with anti-CTLA4 or anti-TIM-3 sensitized tumors to these antibodies without toxicity, suggesting that YST-OVH created inflamed tumors. Moreover, our results support the concept that both the activation of immune responses and the prevention of immunosuppressive responses are essential for achieving therapeutic benefit.

Interestingly, the observed increase in the therapeutic efficacy of YST-OVH was thus likely mediated by enhanced activation of lymphocytes through the PD-1/PD-L1 axis mediated at the virus-treated tumor site, with resultant enhancement in abscopal function. These results mirror previous findings from our group,^22^ demonstrating that YST-OVH expressing hPD-1 blockers (hPD-1scFv) was able to significantly enhance systemic antitumor immunity, an effect that was associated with the expansion and enhancement of the effector and memory functions of tumor-infiltrating CD8^+^ lymphocytes, and a reduction in exhausted PD-1^hi^ CTLA-4^+^ TIM-3^+^ CD8^+^ lymphocytes.

While immunotherapy leads to potent antitumor efficacy, it also leads to immune-related adverse events in patients with cancer. These toxicities stem from unwanted systemic immune activation.^39^ Intratumoral immunotherapy with an OV expressing hPD-1 blockers can be an effective strategy to treat advanced cancer. However, the safety concerns of this ‘arming combination strategy’ and the neurovirulence of HSV-1-based vectors should be thoroughly considered, as treatment-related adverse events have been a serious concern in clinical application. We observed that hPD-1scFv expression in the tumors of YST-OVH-treated mice was much higher than that in the serum of YST-OVH-treated mice, which may minimize systemic side effects. Nevertheless, the favorable outcomes of toxicity studies in humanized mouse models and nonhuman primates should encourage the clinical translation of YST-OVH virotherapy.

There are some limitations to this study that we should be further addressed. First, the in vivo evaluation of the efficacy of OVs armed with an immunomodulator is complicated by the relatively low permissivity for HSV-1 in mouse cancer cells.^40^ The inherent poor replication of YST-OVH in murine cancer cells may result in a low level of hPD-1scFv production in mouse tumors in vivo. It would be intriguing to determine whether the antitumor activity is dependent on the abundance of hPD-1scFv within the TME. Although this issue was not fully investigated in the current study, we speculated that adequate viral replication within tumors might facilitate hPD-1scFv expression and thereafter promote stronger immunomodulatory effects. In addition, YST-OVH treatment offered transient expression of the hPD-1scFv protein due to virus clearance from OV-induced immune response, which may consequently limit the oncolytic and immunotherapeutic activity of virotherapy. Second, although we demonstrated immune status changes in the TME triggered by hPD-1scFv, focusing on the characterization of CD8^+^T cell subsets and Treg cells, it is important to further investigate the behavior and roles of other intratumoral cells, including CD4^+^ T cell subsets, antigen-presenting cells, and TAMs. Due to the limited staining markers used in the current CyTOF analysis, more detailed immune profiling analysis and T cell receptor repertoire analysis need to be performed in the future. Third, it is necessary to elucidate what factors in the cancer-immune set point are expected to influence the sensitivity to this virotherapy by developing panels of biomarkers. Further optimization of the combination strategies for the application of this virotherapy with immune checkpoint blockade may be important for improving clinical efficacy. Apart from arming with immune checkpoint inhibitors, whether the integration of other cytokines or therapeutic molecules into parental OVH may also help to overcome adaptive immune resistance and to further improve the therapeutic efficacy of YST-OVH in the late-stage tumor model is currently under investigation. Lastly, our preliminary data indicated that intratumoral treatment of OVs was more likely to induce anti-tumor memory CD8^+^ T cells but not antiviral memory CD8^+^ T cells at the early stage after virus treatment. As the virotherapy continues, virus treatment can produce more anti-viral memory CD8^+^ T cells,^41–43^ it is reasonable to assume that these cells may promote the clearance of OVs and thereafter limit the oncolytic and immunotherapeutic activity of virotherapy. However, the roles of anti-viral memory CD8^+^ T cells on counteracting the anti-tumor effects of virotherapy remain poorly understood and warrant further investigation.

In summary, these findings serve as a proof of principle that localized immunotherapy with an OV can upregulate the immune-promoting set point (eg, IFN-γ^+^ or GZMB^+^CD8^+^ T cells, CD44^+^CD8^+^ T cells) and immunosuppressive set point (eg, CTLA-4^+^TIM-3^+^CD8^+^ T cells and CTLA^+^ Treg cells and PMN-MDSCs) and that targeting of these pathways through both systemic and localized approaches may be required for optimal therapeutic efficacy. We demonstrate that YST-OVH is an attractive agent for localized immunomodulation with immune checkpoint blockade to enhance the immune response to cancer and provide a strong rationale for further clinical evaluation of this novel therapeutic approach.

## Data Availability

All data relevant to the study are included in the article or uploaded as online supplemental information.
