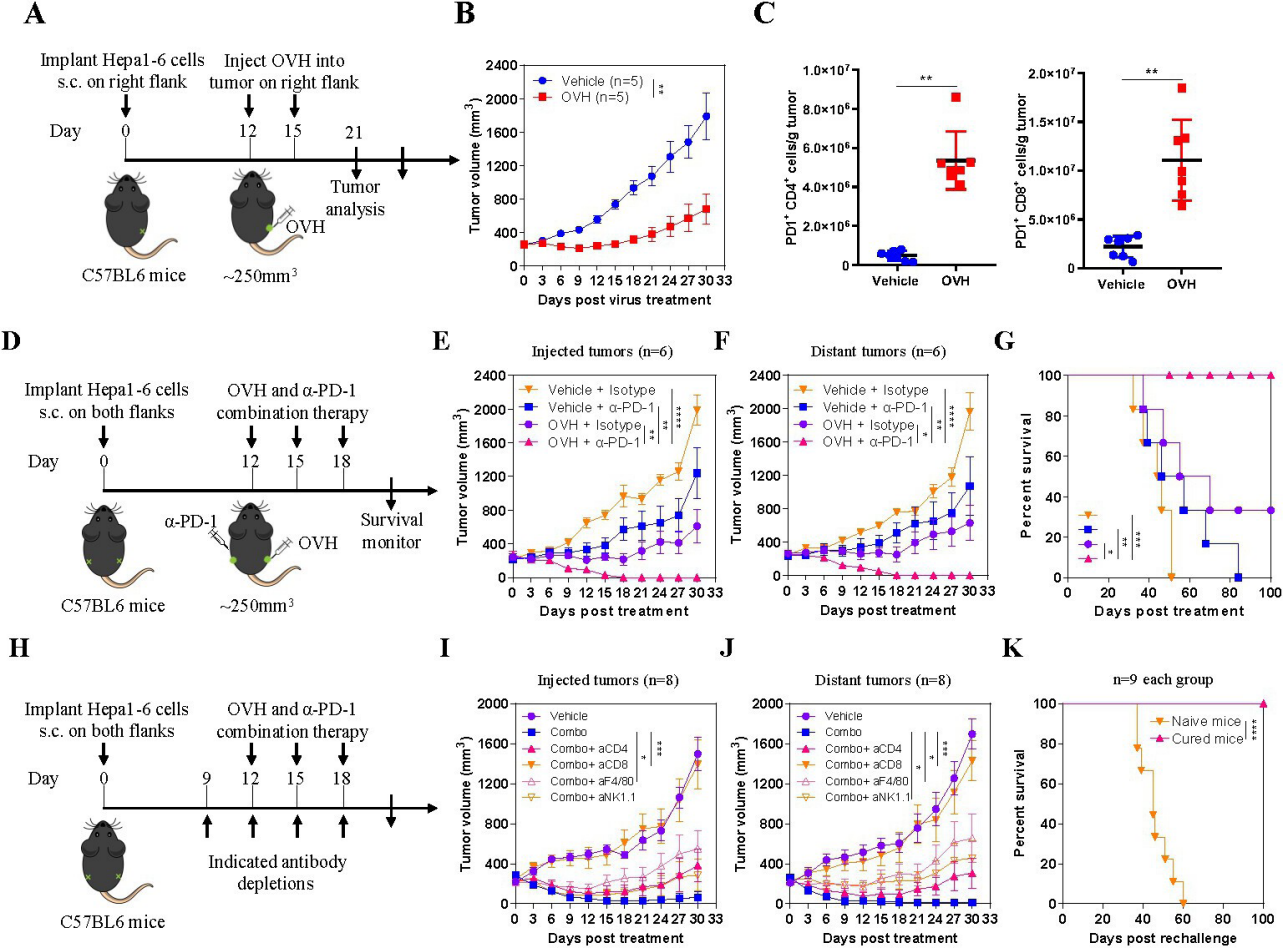# Correction: Oncolytic virus expressing PD-1 inhibitors activates a collaborative intratumoral immune response to control tumor and synergizes with CTLA-4 or TIM-3 blockade

**DOI:** 10.1136/jitc-2022-004762corr1

**Published:** 2024-01-30

Ju F, Luo Y, Lin C, et al. Oncolytic virus expressing PD-1 inhibitors activates a collaborative intratumoral immune response to control tumor and synergizes with CTLA-4 or TIM-3 blockade. *J ImmunoTher Cancer* 2022;10:e004762. doi: 10.1136/jitc-2022-004762

In figure 1K, the legend has been amended to the icon ∇ for ‘Naïve mice', and to the icon ∆ for ‘Cured mice’.