# Supplementary material for: Oncolytic virus expressing PD-1 inhibitors activates a collaborative intratumoral immune response to control tumor and synergizes with CTLA-4 or TIM-3 blockade
Source: J Immunother Cancer. 2022 Jun 10;10(6):e004762. doi: 10.1136/jitc-2022-004762 (PMC9189843; doi:10.1136/jitc-2022-004762)
Supplement: Supplementary data [file jitc-2022-004762supp001.pdf]

Supplementary material

Supplementary methods

Cells

293T, U-2 OS, MDA-MB-453, MDA-MB-231, PC-3, HepG2, Hep3B, PLC/PRF/5, NCI-H1975, NCI-H358, NCI-H520, A549, SW480, HEp-2, BxPC-3, U-87 MG, Saos-2, HFF-1, Caov-3, HeLa, SK-OV-3, Hepa1-6 and 4T1 cells were obtained from the American Type Culture Collection (ATCC). HCT116, CEN-2, Huh-7, SMMC-7721, FaDu, HT-29, H1299 and MCF-7 were obtained from the China Infrastructure of Cell Line Resources. Primary human umbilical vein endothelial cells (HUVECs) were purchased from ScienCell and cultured according to the manufacturer's instructions. Jurkat-Lucia™ NFAT cells were purchased from ScienCell and cultured according to the manufacturer's instructions. 293T, U-2OS, Huh-7, Hepa1-6, MDA-MB-453, MDA-MB-231, SW480, A549, HFF-1 and Caov-3 was maintained in Dulbecco's modified Eagle's medium (DMEM) containing 10% fetal bovine serum (FBS), 100 U/ml penicillin, and 100 µg/ml streptomycin. HepG2, Hep3B, HEp-2, PLC/PRF/5, U-87MG, FaDu, MCF-7 and HeLa were maintained in Eagle's Minimum Essential Medium (EMEM) containing 10% fetal bovine serum (FBS), 100 U/ml penicillin, and 100 µg/ml streptomycin. Saos-2, HCT116, HT-29 and SK-OV-3 were maintained in McCoy's 5A Medium containing 10% fetal bovine serum (FBS), 100 U/ml penicillin, and 100 µg/ml streptomycin. Other cell lines were grown in RPMI-1640 supplemented with 10% fetal bovine serum (FBS), 100 U/ml penicillin, and 100 µg/ml streptomycin. 293T/hPD-1 or 293T/hPD-L1 cells were generated by transfection of pLenti-hPD1 or pLenti-hPD-L1 and selected with 1µg/ml puromycin, gene expression of hPD-1 was confirmed by flow cytometry (FCM), gene expression of hPD-L1 was confirmed by immunoblotting for hPD-L1. Hepa1-6-luc cells were generated by transduction with a lentiviral vector encoding luciferase. Hepa1-6-luc clones were selected in the culture medium containing 1 µg/ml puromycin, gene expression of luciferase was confirmed by Bright-Glo™ Luciferase Assay (Promega). Hepa1-6-OVA cells were generated as previously described.[1] All cell lines were maintained at 37°C and 5% CO_2.

Antibodies and reagents

Polyclonal antibodies including anti-β-Actin antibody C4 and anti-VP5 antibody 6F10 were from Santa Cruz. Polyclonal anti-ICP34.5 antibodies were produced as previously described.[2] An anti-ICP0 monoclonal antibody (mAb) 5H7 and an anti-gD mAb DL6 were from Santa Cruz, An HRP-conjugated His-Tag mAb (anti-His-HRP) was from Proteintech. Antibodies used for flow

cytometry were purchased from Biolegend (CD45 Brilliant Violet 605, CD8a FITC, TNF-α PE, Granzyme B PE/cy7 and their corresponding isotype control antibodies), and BD Pharmingen (IFN-γ APC). All checkpoint antibodies and isotype control antibodies were purchased from BioXCell unless otherwise indicated. Pembrolizumab and Nivolumab were purchased from MCE.

PD-1 antibody production

Six-week-old female BALB/c mice were maintained in individually ventilated cages under specific pathogen free condition and immunized with 1×10^7 293T/hPD-1 cells in 500 µl of PBS at multiple sites by standard vaccination procedures. The hybridoma generation procedure followed the protocol reported previously.[3] The positive clones were selected by an indirect ELISA assay coated with recombinant human PD-1 protein, and subjected to colonized culture for obtaining stable single clones. MAbs were prepared by ascites production and purified by using protein G chromatography according to the manufacturer's instructions.

Antibody evaluation

The binding ability of full-length anti-PD-1 antibodies and hPD-1scFv was determined by an indirect chemiluminescence immunoassay (CEIA). Briefly, 96-well plates were coated with 10 ng/well of hPD-1-His protein (Sino Biological), and nonspecific binding was blocked with PBS containing 5% BSA. Purified anti-PD-1 antibodies, hPD-1scFv, supernatants from YST-OVH-infected or non-infected cells, or serum from YST-OVH-treated or non-treated mouse were added to the wells for 1 h incubation, followed by washing and reaction with anti-His-HRP antibody (Proteintech). After the addition of Luminol substrates (Wantai BioPharm) for 5 min, the plates were measured with a chemiluminescence reader (Berthod). For detection of hPD-1scFv in tumors, the tumors were homogenized with PBS by gentleMACS tissue dissociator (Miltenyi BioTec) according to the manufacturer's instructions, and the lysates were assayed using the above-mentioned method. For quantitative detection of hPD-1scFv, a serial dilution of purified hPD-1scFv was used to generate a standard curve.

The blocking activity of full-length anti-PD-1 antibodies and hPD-1scFv was determined using a blocking CEIA assay. Briefly, 96-well plates were coated with 100 ng/well of hPD-1-His protein, and nonspecific binding was blocked with PBS containing 5% BSA. Purified hPD-1scFv or anti-PD-1 antibodies were first diluted from 10 µg/ml in PBS containing 20% CBS, followed by two-fold serial dilutions to eight gradients. Then, each dilution together with 100 ng/well biotinylated hPD-L1-Fc (Sino Biological) were added to the wells for 1 h incubation, followed by washing,

reaction and detection. The inhibitory ratio was calculated as follows: %inhibitory = 100× (1− (average value for each dilution/average value for control)). The results were analyzed by nonlinear, dose-response regression analysis using GraphPad Prism 7 software.

RNA isolation and gene amplification

Total RNA was extracted with TRIzol Reagent (Invitrogen) and reverse-transcribed into cDNA with random primers by SuperScript® III Reverse Transcriptase (Invitrogen). The cDNA products were then used as templates for PCR amplification of the variable regions of the heavy chain and light chain with KOD-Plus polymerase (TOYOBO) using an Ig-Prime kit (Novagen). The variable sequences of the heavy chain and light chain were sequenced and cloned into pFUSE antibody cassettes (InvivoGen) for further development.

Humanization of the anti-PD-1 antibody 17D5

Humanization of an anti-PD-1 antibody by CDR grafting and combinatorial library screening was performed as previously described.[4] Humanized antibodies were first evaluated by testing their binding reactivity with a recombinant hPD-1 protein and ability to block the interaction between PD-1 and PD-L1 and then examined for their humanness score. 293T cells were used to express humanized anti-PD1 antibodies by transfection of recombinant humanized antibody cassettes in FreeStyle 293 Expression Medium, and hu17D5 was purified by using protein G chromatography according to the manufacturer's instructions.

hPD-1 scFv construction and expression

Humanized anti-PD1 antibody 17D5 (hu17D5) was selected for scFv construction, the fragment encoding scFv against hPD-1 consisted of a secretion signal peptide, light chain, $3\times G_4S$, heavy chain and 8×His tag, which were first amplified from hu17D5 cDNA and sequentially assembled into pTT5 vector (CNRC) under the control of the human cytomegalovirus promoter. 293T cells were used to express hPD-1scFv by transfection of PTT5-hPD-1scFv in FreeStyle 293 Expression Medium, hPD-1scFv was purified by using Ni-NTA chromatography (GE Healthcare) according to the manufacturer's instructions. hPD-1scFv was eluted using 250 mM Imidazole and dialyzed in PBS.

T cell activation assays

The T cell-activating activity of full length anti-PD-1 antibodies and hPD-1scFv was determined using a PD-L1 overexpressing cells and PBMCs co-culture assay. Human PBMCs of a healthy donor (5×10^4 per well) (Lonza) were stimulated with anti-CD3/CD28-coated beads (Miltenyi) or

cultured without stimulation for 2 days in a 96-well plate. Then, 1×10^4 293T/hPD-L1 cells and 0.001 to 10 μg/ml of anti-PD-1 antibodies and hPD-1scFv were added and cultured at 37℃ for 3 days. The IL-2 levels in the supernatant were assayed by Human IL-2 Quantikine ELISA Kit (R&D Systems).

Co-culture of tumor cells with Jurkat cells

1×10^4 irradiated MDA-MB-231 cells and 5x10^3 Jurkat-Lucia™ NFAT cells were seeded into wells. After 30 minutes, anti-CD3 (1 μg/ml), anti-CD28 (1 μg/ml) and hPD-1scFv were added to the culture systems. After 24 h of coculture, luciferase activities were measured using the Bright-Glo™ Luciferase Assay (Promega) and a chemiluminescence reader (Berthod) according to the manufacturer's protocol.

Cytotoxicity assay

Cells were seeded in 96-well plates at 1×10^4 cells per well in 100 µl of complete medium and infected with the indicated virus or mock-infected. After treatment for 72 h, 10 µl of CCK-8 (MCE) was added to the cells, and cells could grow at 37°C for another 4 h. The optical absorbance was determined at 450 nm using a microplate reader (Thermo Scientific).

Western blot analysis

Cells were lysed in RIPA lysis buffer (Thermo Scientific) containing protease inhibitor cocktail (MCE). Cell lysates were separated by SDS-PAGE and transferred onto a nitrocellulose membrane. After membranes were blocked with blocking buffer containing 5% BSA for 1 h, they were probed with the indicated primary antibodies overnight at 4℃, followed by incubation with HRP-conjugated secondary antibodies for 1 h at room temperature, and finally visualized with the Lumi-Light^{PLUS} Western blotting Substrate (Roche).

Tumor-infiltrating lymphocytes (TILs) isolation and analysis

For isolation of TILs, mice were sacrificed and the tumors were harvested on days 7 after two doses of the indicated treatment. Tumors were dissected and dissociated with lysis buffer (1 mg/ml Collagenase D (Sigma) and 100 μg/ml DNase I (Sigma) in RPMI1640 medium with 2% FBS) for 2 h with continuous agitation. Digestion mixture were homogenized by repeated pipetting and filtered through a 70-μm filter. Cell suspensions were first stained with Zombie Aqua™ Fixable Viability Kit to eliminate dead cells, stained with the corresponding antibodies, incubated for 30 minutes at 4°C, and then subjected to FCM analysis.

Depletions

Depleting anti-CD8 antibodies (clone 53.6.72), anti-CD4 antibodies (clone GK1.5), anti-NK1.1 antibodies (clone GK1.5), anti-F4/80 antibodies (clone GK1.5) and isotype control antibodies were obtained from BioXCell. 400 μg depleting antibodies or rat IgG isotype control antibodies were intraperitoneally injected 3 days before virus treatment, and then repeatedly injected on days 0, 3 and 6 after injection. 10^7 PFU of virus was intratumorally injected and 200 μg of anti-PD-1 was intraperitoneally injected when the tumors reached 250 mm^3, and then repeatedly injected on days 3 and 6 after the initial treatment. Tumor size was monitored for 30 days.

RNA-seq analysis of tumors

Tumor cells (5×10^6) in 100 μl of PBS were subcutaneously inoculated into the right flank of PD-1-HU mice and allowed to establish for 6 days. Once the tumors reached the indicated volume, 10^7 PFU of virus and/or 200 μg anti-CTLA-4 were injected and then repeatedly injected on days 3 and 6 after the initial treatment. On day 3 after the last treatment, the tumors were harvested and stored at -80°C. Biological triplicates for each group were used to extract total RNA using an RNeasy kit (Qiagen), and RNA quality was measured using an Agilent 2100 Bioanalyzer (Agilent Technologies). One microgram of total RNA with an RIN value above 6.5 was used for subsequent library preparation. Then, libraries with different indices were multiplexed and loaded on an Illumina HiSeq instrument according to the manufacturer's instructions (Illumina). Sequencing was carried out using a 2×150 bp paired-end (PE) configuration; image analysis and base calling were conducted by HiSeq Control Software (HCS) + OLB + GAPipeline-1.6 (Illumina) on a HiSeq instrument. RNA-seq analysis was performed by GeneWiz. The change in gene expression was calculated for each gene in relation to an untreated control. GO enrichment analysis was performed using ClusterProfiler, and the top 10 most enriched pathways are shown in a plot. Heatmaps of normalized expression values of the enriched gene clusters identified by GO analysis were generated using the R package pheatmap.

Histology

For hematoxylin and eosin (H&E) staining, tissues were fixed in 10% formalin, embedded in paraffin, and stained with H&E. Then the sections were washed, and mounted with cover slips. Images were captured with a research-level upright microscope (Olympus).

Cell profiling using CyTOF

Single-cell suspensions of TILs were prepared as described previously. Tumors were mechanically disrupted and digested for 2 h at 37°C in lysis buffer. The released cells were pelleted, resuspended

in 3 ml of ACK Lysing Buffer (Thermo Fisher Scientific) for 2 min to lyse red blood cells at room temperature and then filtered through a 70-μm cell strainer. TILs and tumor cells were separated using Percoll gradient centrifugation. Immune cells were enriched from the 40%/70% Percoll interface. For the CyTOF assay, unconjugated antibodies (Table S5) were obtained from Fluidigm and conjugated in-house using the Maxpar R X8 Multimetal Labeling Kit (Fluidigm) according to the manufacturer's instructions. Briefly, single-cell suspensions were stained with 1 μM cisplatin (Fluidigm) for 15 min and then blocked with Fc receptor blocking buffer (Biolegend) for 10 min at room temperature. Cells from each sample were incubated with a metal-conjugated surface antibody cocktail on ice for 30 min and barcoded with a unique combination of palladium metal barcodes according to the manufacturer's instructions (Fluidigm). Next, the cells were pooled together, followed by fixation and permeabilization using the Nuclear Staining Buffer Set (Fluidigm). The cells were subsequently stained with a metal-conjugated intracellular antibody cocktail for 30 min at 4°C. After washing, the cells were incubated in 1 ml of intercalator buffer (0.125 nM MaxPar Intercalator-Ir in 1 ml of Fixation & Permeabilization Buffer). Prior to acquisition, the cells were diluted to 8×10^5 cells/ml in deionized water containing 10% EQ Four Element Calibration Beads (Fluidigm) and filtered through a 70-μm nylon filter. Events were acquired on a CyTOF 2 Helios upgraded mass cytometer at an event rate of 200-300 cells/second at the Flow Cytometry and Cellular Imaging Facility of Xiamen University. .fcs files were normalized to the EQ 4-element bead signal (Lot P15K0802, Passport EQ 4_P13H2302) in 100-s interval windows using normalization software (version 6.7.1014, Fluidigm). Mass tag barcodes were resolved with a doublet filtering scheme using Debarcoder (Fluidigm). Live immune cells were manually gated with FlowJo by event length, live/dead discrimination, and the expression status of CD45. The data were then exported for downstream analysis and transformed with a coefficient of 5 with the cytofAsinh method. For downstream analyses, individual sample data were subsampled to 10000 events of the $CD45^+$ population. Contour plots were used to specifically define the T cell clusters in manual gates with FlowJo and exported as .fcs files. t-SNE dimensionality reduction and PhenoGraph clustering analyses were performed using the tool cytofkit run in R package software.[5] Partial markers were used during the t-SNE[6] and PhenoGraph analyses.[7] For the generation of heatmap displays, marker expression was normalized by dividing by the range of all markers (expression range from the 1st to 99th percentile). Data displays were generated using the ggplot2 R package.[8]

Safety study

For the evaluation of the neurovirulence of YST-OVH, twelve 3- to 4.5-year-old male or female rhesus macaques were obtained from the Beijing Prima Biotech (Beijing, China) before the beginning of the study. Animals were housed and maintained at the animal facility of Beijing JOINN Laboratories according to guidelines provided by the Institutional Animal Care and Use Committee for animal welfare. Two animals were randomly selected for inclusion in the control group, and the other ten animals received one left thalamic injection (i.c.) and one right i.c. injection of virus at a dose of 1.65×10^7 PFU/injection. The i.c. inoculations were performed with a stereotaxic instrument (Stoelting). Animals were monitored for 22 days following inoculation. Finally, the treated animals were euthanized with pentobarbital (200 mg/kg, i.p.), followed by histological examinations of the injection site, frontal lobe, parietal lobe, temporal lobe, occipital lobe, hippocampus, thalamus, midbrain, pons, cerebellum, medulla, and spinal cord (neck, chest, and lumbar).

For the evaluation of the toxicity study of YST-OVH, twenty 3- to 5-year-old male or female cynomolgus monkeys were obtained from Guangxi Xiongsen Non-human Primates Laboratory (Guangxi, China) before the beginning of the study. Animals were housed and maintained at the animal facility of Beijing JOINN Laboratories. Animals were randomized into two groups (n=10 for each group), and each group included 5 male and 5 female cynomolgus monkeys. YST-OVH was administered by i.v. injection at a dose of 2.4×10^8 PFU in 3 ml of saline for three cycles of a total of 15 injections on days 1-5, days 13-17, and days 25-29 relative to the start date. Six monkeys from the YST-OVH-treated or untreated group were euthanized with pentobarbital (200 mg/kg, i.p.) on day 30, followed by histological examinations. The remaining animals were monitored for 58 days following inoculation. Animals were bled on days -3, 6, 25, 30 and 58 relative to the start date for a total of five blood draws. Clinical symptoms, body weight and body temperature were monitored on days -14, -3, 5, 12, 19, 26, 30, 33, 40, 47, 54, 57 and 58 relative to the start date. Determination of serum biochemical parameters was performed on a TOSHIBA TBA-120FR Chemistry Analyzer and EasyLyte ® electrolyte analyzer. Determination of hematological parameters were performed on a Siemens Advia 2120 Hematology Analyzer. Determination of cytokines was performed on a BD FACSCalibur using a CBA nonhuman primate Th1/Th2 cytokine kit (BD), according to the manufacturer's instructions. The reference values (95% CIs)

for the hematological and biochemical parameters were based on observations of 2000 Cynomolgus monkeys (half male and half female).

References:

1 Lin C, Ren W, Luo Y*, et al.* Intratumoral Delivery of a PD-1-Blocking scFv Encoded in Oncolytic HSV-1 Promotes Antitumor Immunity and Synergizes with TIGIT Blockade. *Cancer Immunol Res* 2020;8:632-47.

2 Luo Y, Lin C, Zou Y*, et al.* Tumor-targeting oncolytic virus elicits potent immunotherapeutic vaccine responses to tumor antigens. *Oncoimmunology* 2020;9:1726168.

3 Chen Q, Qiu S, Li H*, et al.* A novel approach for rapid high-throughput selection of recombinant functional rat monoclonal antibodies. *BMC Immunol* 2018;19:35.

4 Wu H, Nie Y, Huse WD*, et al.* Humanization of a murine monoclonal antibody by simultaneous optimization of framework and CDR residues. *J Mol Biol* 1999;294:151-62.

5 Chen H, Lau MC, Wong MT*, et al.* Cytofkit: A Bioconductor Package for an Integrated Mass Cytometry Data Analysis Pipeline. *PLoS Comput Biol* 2016;12:e1005112.

6 van der Maaten L, Hinton G. Visualizing data using t-SNE. *J Mach Learn Res* 2008;9:2579–605.

7 Levine JH, Simonds EF, Bendall SC*, et al.* Data-Driven Phenotypic Dissection of AML Reveals Progenitor-like Cells that Correlate with Prognosis. *Cell* 2015;162:184-97.

8 Xu L, Zhu Y, Chen L*, et al.* Prognostic value of diametrically polarized tumor-associated macrophages in renal cell carcinoma. *Ann Surg Oncol* 2014;21:3142-50.

9 Laffins MM, Mellal N, Almlie CL*, et al.* Evaluation of Infrared Thermometry in Cynomolgus Macaques (Macaca fascicularis). *J Am Assoc Lab Anim Sci* 2017;56:84-89.

10 Poole TB, English P, Universities Federation for Animal Welfare. *The UFAW handbook on the care and management of laboratory animals*, 7th edn. Blackwell Science (1999).

Supplementary figures

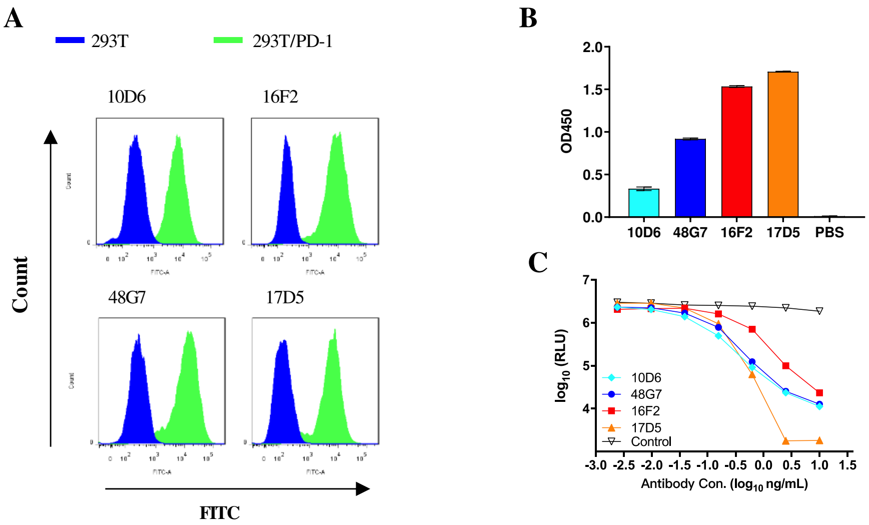

Fig. S1. Screening of mouse anti-PD-1 antibodies. (A) Flow cytometric analysis of the reactivity of anti-PD-1 antibodies against 293T/PD-1 cells. (B) The reactivity of anti-PD-1 antibodies against the human PD-1 protein was determined by indirect ELISA. (C) The blocking activity of anti-PD-1 antibodies was determined by a blocking CEIA.

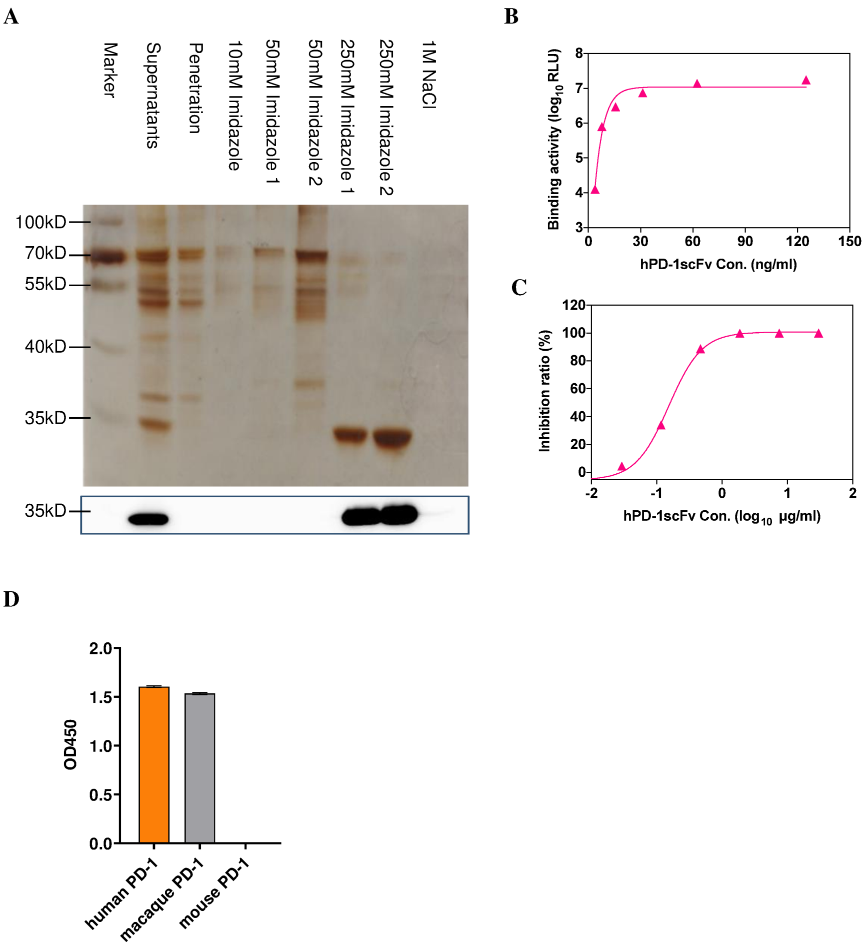

Fig. S2. Characteristics of hPD-1scFv proteins. (A) Purification of hPD-1scFv proteins. (B) The reactivity of hPD-1scFv against a his-hPD-1 protein was determined by an indirect CEIA. (C) The blocking activity of hPD-1scFv was determined by a blocking CEIA. (D) The reactivity of anti-PD-1 antibodies against human, macaque, and mouse PD-1 proteins was determined by indirect ELISA.

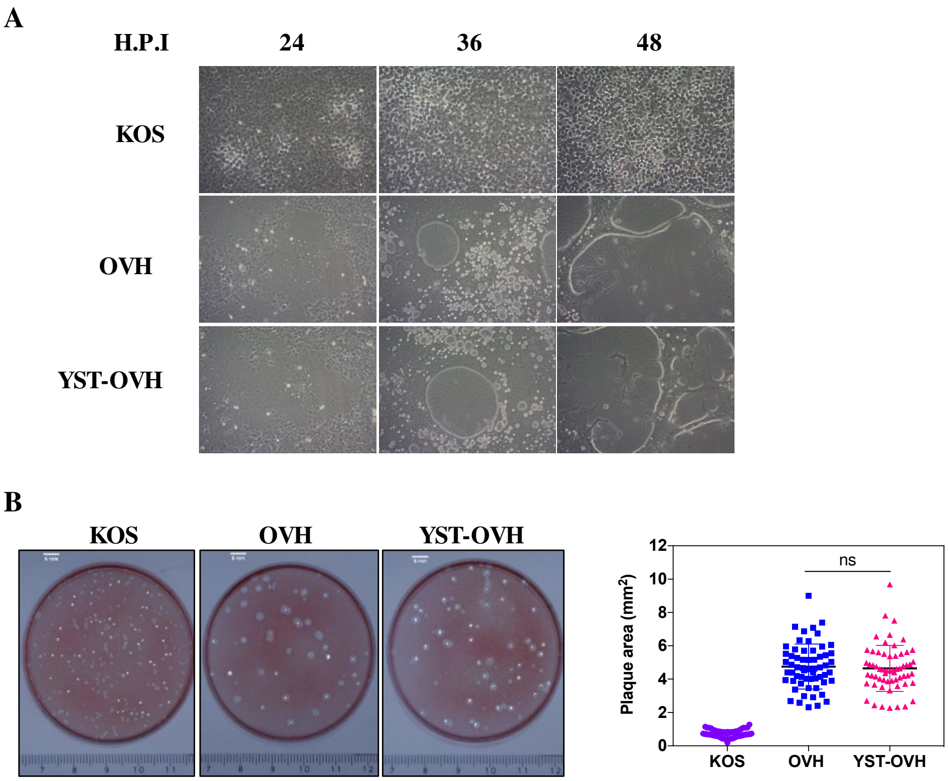

Fig. S3. Characteristics of YST-OVH tested on U-2 OS cells. (A) Infection of U-2 OS cells with KOS, OVH or YST-OVH at different timepoints. H.P.I: hours post-infection. (B) Plaque assays for KOS, OVH and YST-OVH on U-2 OS monolayers. Sixty random plaques were selected from each virus-infected dish, and the average area of individual plaques was calculated. Data are shown as the means ± SEM; unpaired two-sided Student's t test (B).

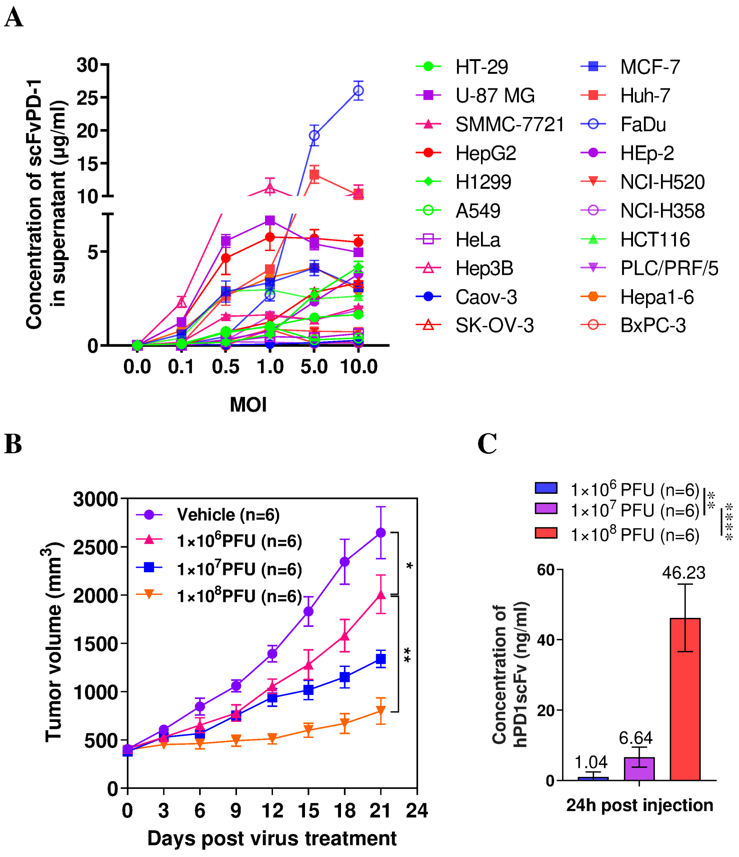

Fig. S4. Expression of hPD-1scFv in vitro and in vivo after YST-OVH treatment. (A) Human and murine cancer cells were infected with YST-OVH at the indicated MOIs, and the expression levels of hPD-1scFv in the supernatants were analyzed at 48 h post-infection. (B) Dose-escalation regimens to evaluate the oncolytic effects of YST-OVH on an HCT116 mouse model. (C) Assessment of hPD-1scFv expression in the serum of YST-OVH-treated nude mice at 24 h post-treatment under different dosing schemes. All values are presented as the mean ± SEM; repeated-measure ANOVA (B) or unpaired two-tailed Student's t test (C).

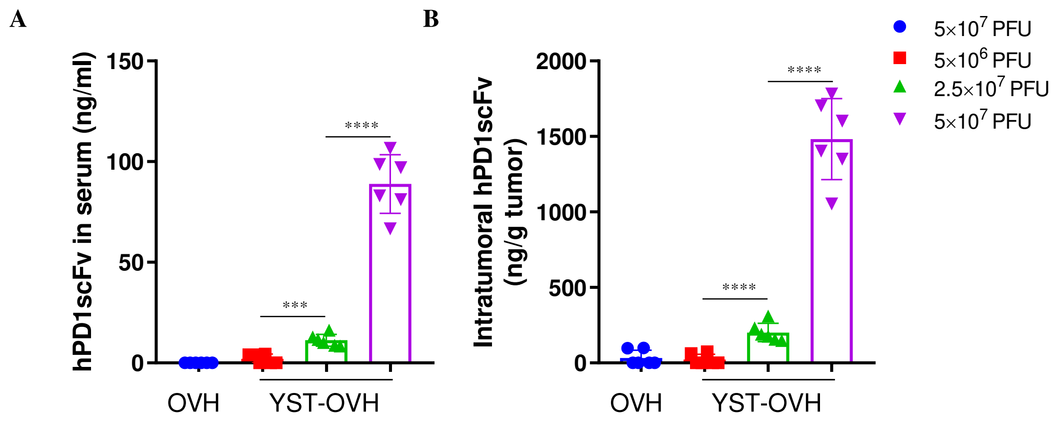

Fig. S5. Assessment of hPD-1scFv expression in the serum (A) and tumors (B) of OV-treated immunocompetent mice at 24 h post-treatment under different dosing schemes. All values are presented as the mean ± SEM; unpaired two-tailed Student's t test (A, B).

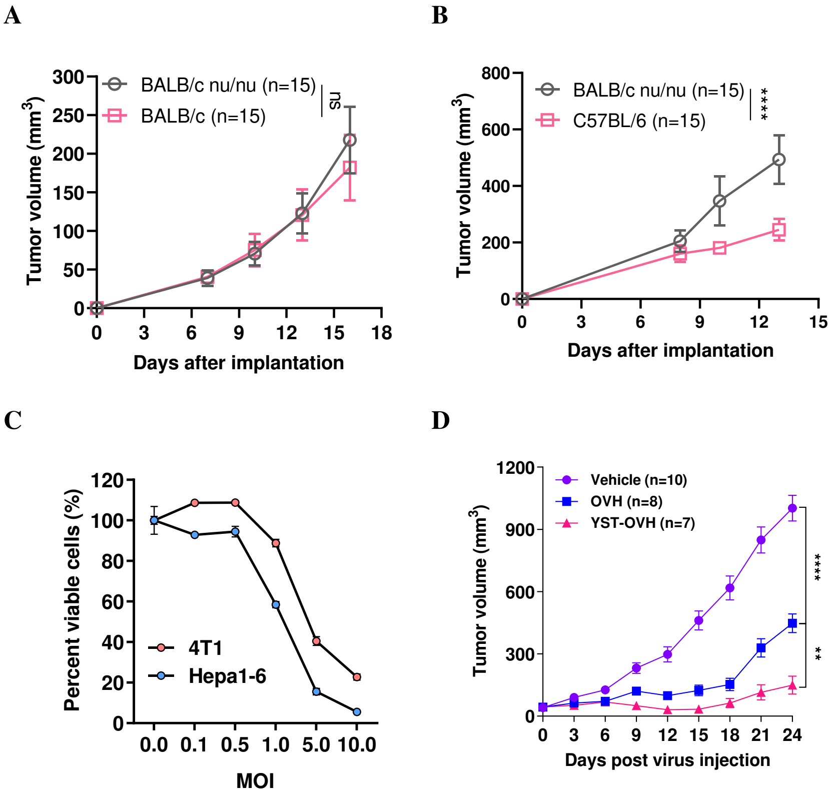

Fig. S6. Intratumoral injection of YST-OVH enhanced antitumor efficacy in a less immunogenic 4T1 tumor model. (A) The tumor growth of 4T1 tumors in immunodeficient nude mice and BALB/c mice is shown. **(B)** The tumor growth of Hepa 1-6 tumors in immunodeficient nude mice and C57BL/6 mice is shown. **(C)** Hepa 1-6 and 4T1 cells were infected with YST-OVH at various MOIs. Cell viability was analyzed at 3 days after infection by a CCK8 cell viability assay. **(D)** 4T1 tumor-bearing PD-1-HU mice received vehicle, OVH or YST-OVH therapy. The tumor growth of injected tumors is shown. All values are presented as the mean ± SEM; repeated-measure ANOVA (A, B, D).

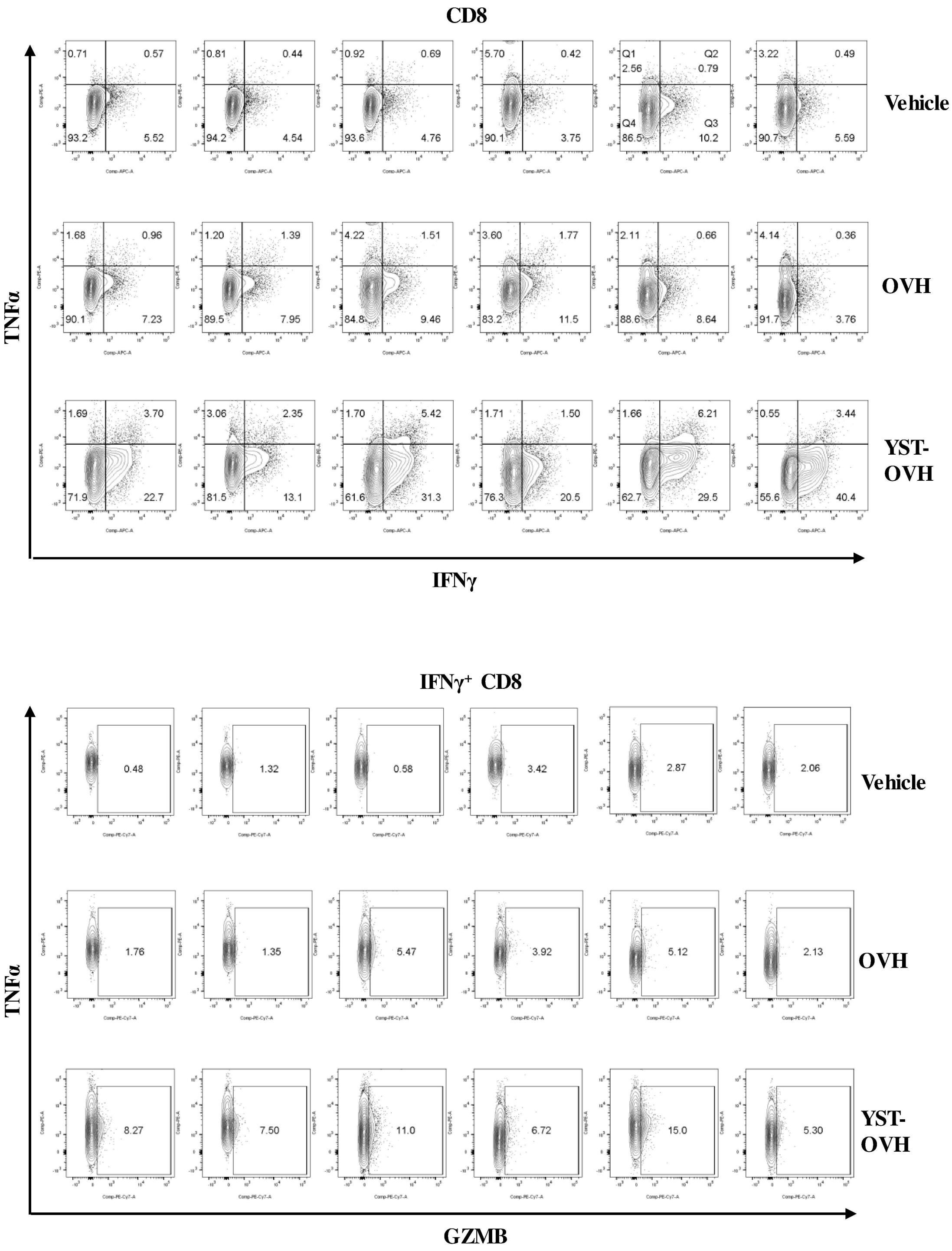
CD8
TNFα
IFNγ
Vehicle
OVH
YST-OVH
0.71
0.57
93.2
5.52
0.81
0.44
94.2
4.54
0.92
0.69
93.6
4.76
5.70
0.42
90.1
3.75
Q1
Q2
2.56
0.79
Q4
Q3
86.5
10.2
3.22
0.49
90.7
5.59
1.68
0.96
90.1
7.23
1.20
1.39
89.5
7.95
4.22
1.51
84.8
9.46
3.60
1.77
83.2
11.5
2.11
0.66
88.6
8.64
4.14
0.36
91.7
3.76
1.69
3.70
71.9
22.7
3.06
2.35
81.5
13.1
1.70
5.42
61.6
31.3
1.71
1.50
76.3
20.5
1.66
6.21
62.7
29.5
0.55
3.44
55.6
40.4
IFNγ+ CD8
GZMB
0.48
1.32
0.58
3.42
2.87
2.06
1.76
1.35
5.47
3.92
5.12
2.13
8.27
7.50
11.0
6.72
15.0
5.30

Fig. S7. Representative flow cytometric plots of IFN-γ$^+$, IFN-γ$^+$TNF-α$^+$ and GZMB$^+$CD8$^+$ T cells in the tumor-infiltrating CD8$^+$ T cell population.

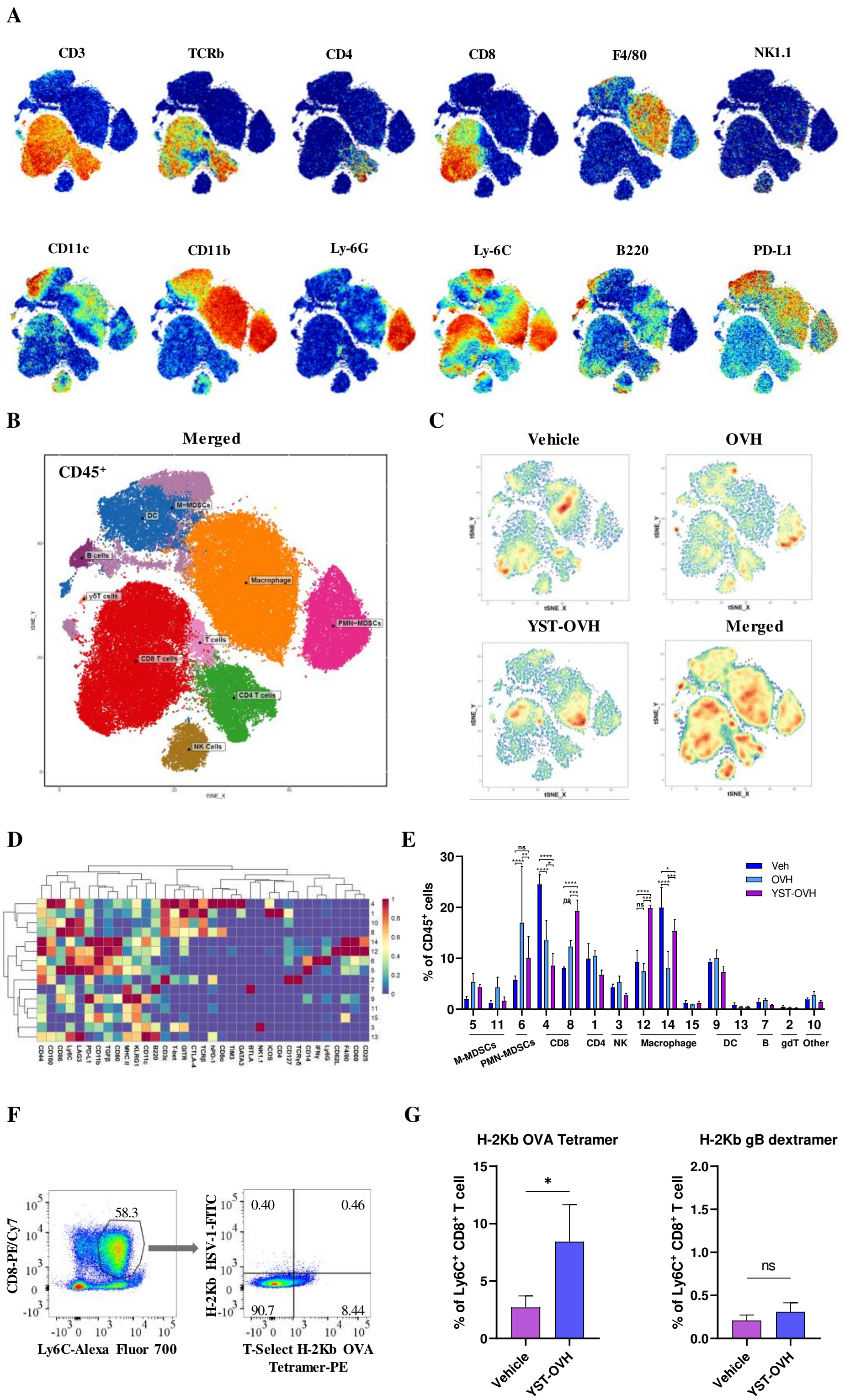
A
CD3
TCRb
CD4
CD8
F4/80
NK1.1
CD11c
CD11b
Ly-6G
Ly-6C
B220
PD-L1
B
Merged
CD45+
C
Vehicle
OVH
YST-OVH
Merged
D
E
% of CD45+ cells
Veh
OVH
YST-OVH
M-MDSCs
PMN-MDSCs
CD8
CD4
NK
Macrophage
DC
B
gdT
Other
F
58.3
0.40
0.46
90.7
8.44
CD8-PE/Cy7
Ly6C-Alexa Fluor 700
H-2Kb HSV-1-FITC
T-Select H-2Kb OVA Tetramer-PE
G
H-2Kb OVA Tetramer
H-2Kb gB dextramer
% of Ly6C+ CD8+ T cell
Vehicle
YST-OVH
ns

Fig. S8. OV treatment modulates Hepa1-6 tumor-infiltrating immune populations. (A) t-SNE plot of $CD45^+$ Hepa1-6 tumor immune infiltrates overlaid with the expression of selected markers. (B) t-SNE plot derived from CyTOF analysis of tumor immune infiltrates obtained from each treatment group. Cells are colored by the clusters identified by PhenoGraph. (C) Density t-SNE plots of equal numbers of tumor immune infiltrates from each treatment group. (D) Heatmap displaying the normalized marker expression of each $CD45^+$ cell cluster. (E) Quantitative analysis of the T cell clusters as a percentage of $CD45^+$ cells. (F) Representative flow cytometry plots show characteristic markers of selected intratumoral T-cell clusters. HSV-1-specific H-2 Kb-HSV-1 gB dextramer, and SIINFEKL-specific H-2Kb-OVA Tetramer. (G) Quantification of antigen-specific and virus-specific memory $CD8^+$ T cells in tumors. Quantitative data are presented as the mean ± SEM and were analyzed by an unpaired two-sided Student's t test.

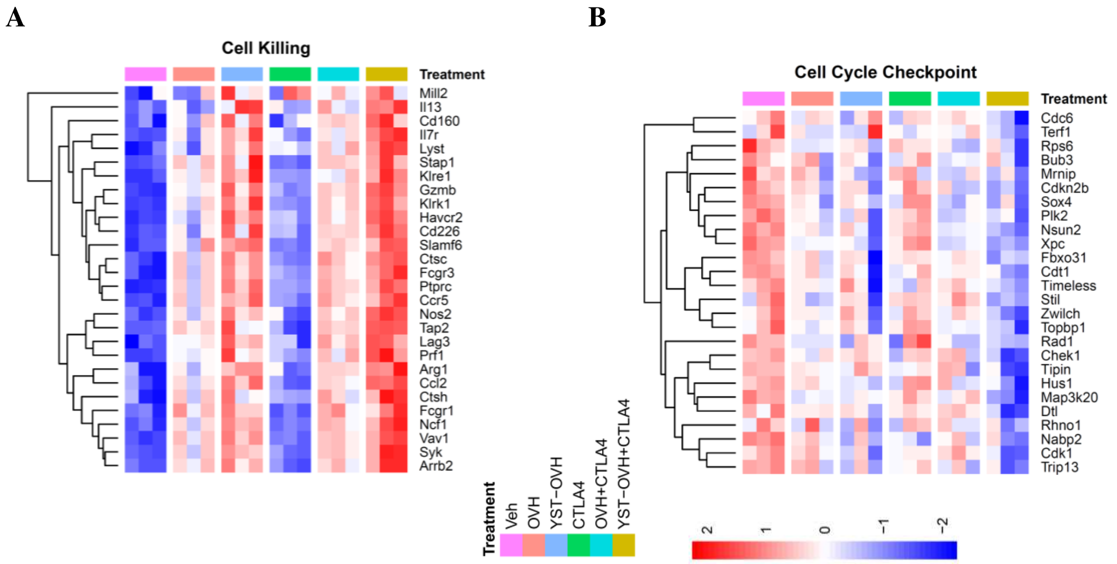

Fig. S9. Heatmap showing the expression of differentially expressed cytotoxicity genes (A) and cell cycle checkpoint genes (B). Gene expression was normalized to values obtained for the untreated control.

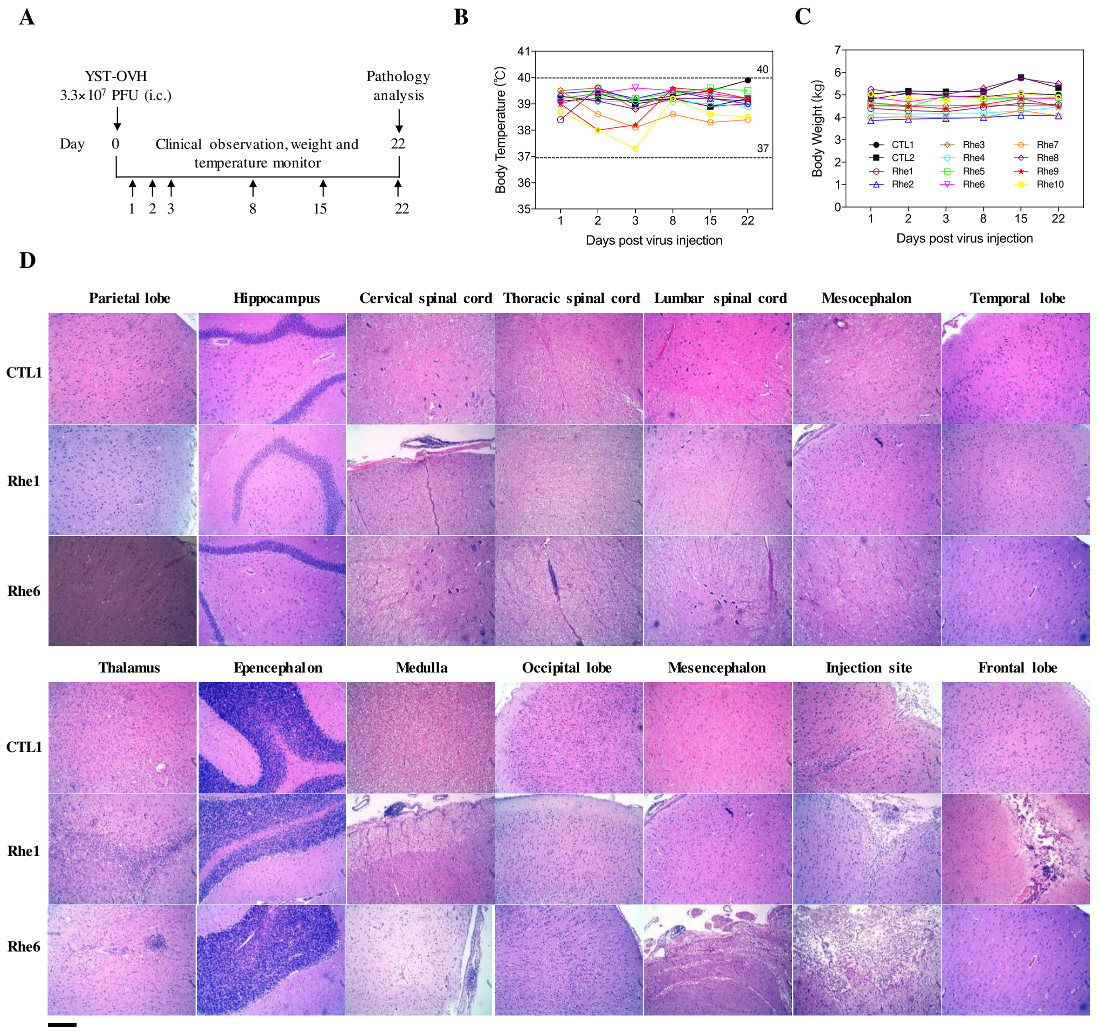

Fig. S10. Intrathalamic inoculation of YST-OVH showed no neurotoxicity in rhesus macaques. **(A)** Timeline of the experimental setup. i.c., intrathalamic. **(B)** Body temperature was recorded at the indicated times. Control, n = 2; YST-OVH, n = 8. Dashed lines represent reference values: 37.0°C to 40°C.[9, 10] **(C)** Animals were weighed at the indicated times. **(D)** Hematoxylin and eosin staining of the indicated tissues. Scale bar, 200 μm.

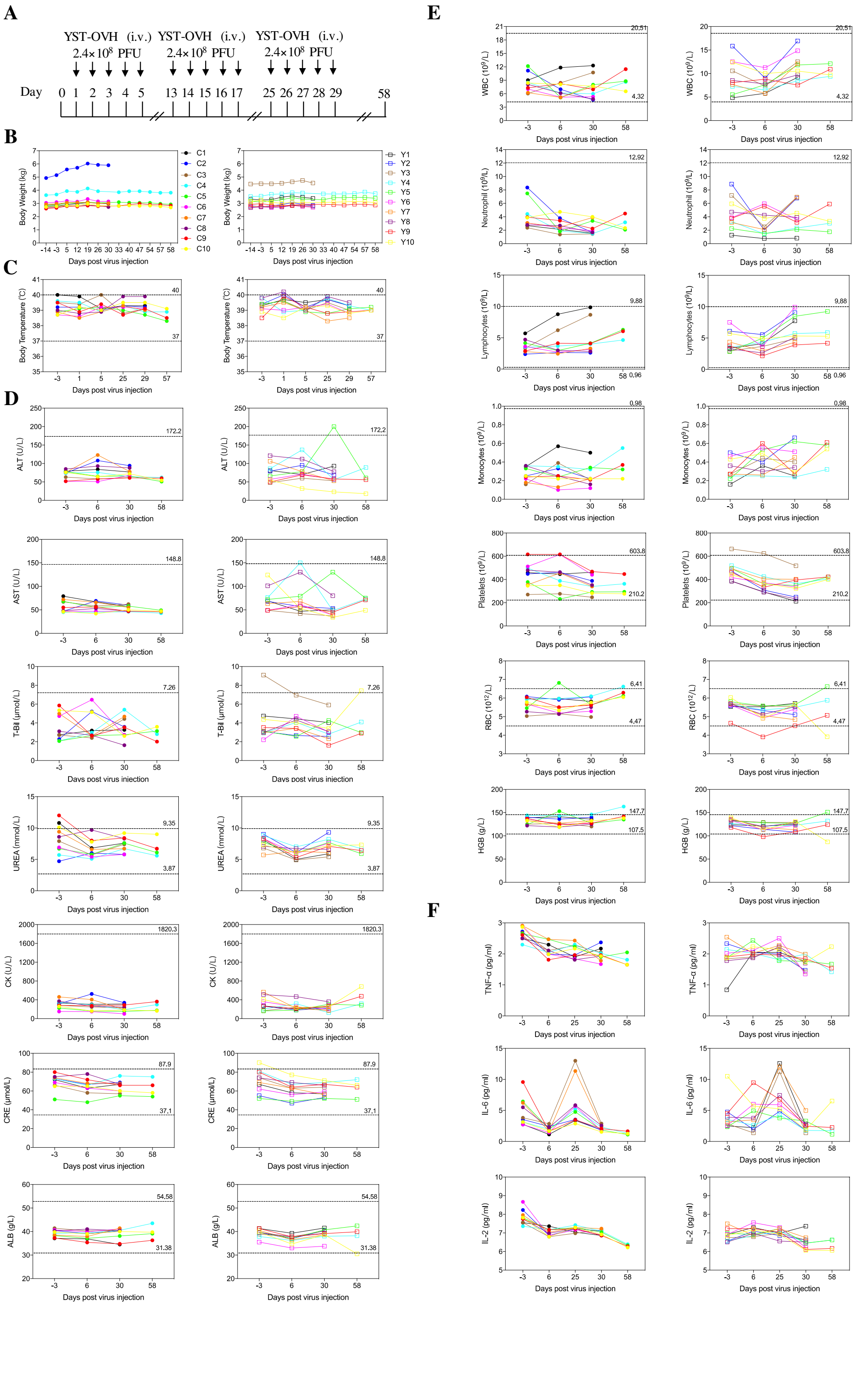
A
YST-OVH (i.v.)
2.4×10⁸ PFU
Day
B
Body Weight (kg)
Days post virus injection
C
Body Temperature (℃)
D
ALT (U/L)
AST (U/L)
T-Bil (μmol/L)
UREA (mmol/L)
CK (U/L)
CRE (μmol/L)
ALB (g/L)
E
WBC (10⁹/L)
Neutrophil (10⁹/L)
Lymphocytes (10⁹/L)
Monocytes (10⁹/L)
Platelets (10⁹/L)
RBC (10¹²/L)
HGB (g/L)
F
TNF-α (pg/ml)
IL-6 (pg/ml)
IL-2 (pg/ml)

Fig. 11. Systemic administration of YST-OVH is well tolerated in cynomolgus monkeys. (A) Timeline of the experimental setup. **(B)** Animals were weighed at the indicated times. Control, n = 10; YST-OVH, n = 10. **(C)** Body temperature was recorded at the indicated times. **(D)** Serum biochemical parameters for animals injected with YST-OVH. The dashed lines represent the reference values of the biochemical parameters: alanine transaminase (ALT), 0 to 172.20 U/L; aspartate aminotransferase (AST), 0 to 148.80 U/L; total bilirubin (T-Bil), 0 to 7.26 μM; blood urea nitrogen (UREA), 0 to 9.35 mM; creatine kinase (CK), 0 to 1820.30 U/L; creatinine (CRE), 37.10 to 87.90 μM; and albumin (ALB), 31.38 to 54.58 g/L. **(E)** Hematological parameters for animals injected with YST-OVH. The dashed lines represent the reference values of hematological parameters: white blood cells (WBCs), 4.32×10^{9} to 20.51×10^{9}/L; platelets, 210.02×10^{9} to 603.80×10^{9}/L; lymphocytes: 0.96×10^{9} to 9.88×10^{9}/L; neutrophils, 0 to 12.92×10^{9}/L; monocytes, 0 to 0.98×10^{9}/L; red blood cells (RBC), 4.47×10^{12} to 6.41×10^{12}/L; and hemoglobin (HGB), 107.50 to 147.70 g/L. **(F)** Plasma cytokine analysis of animals injected with YST-OVH.

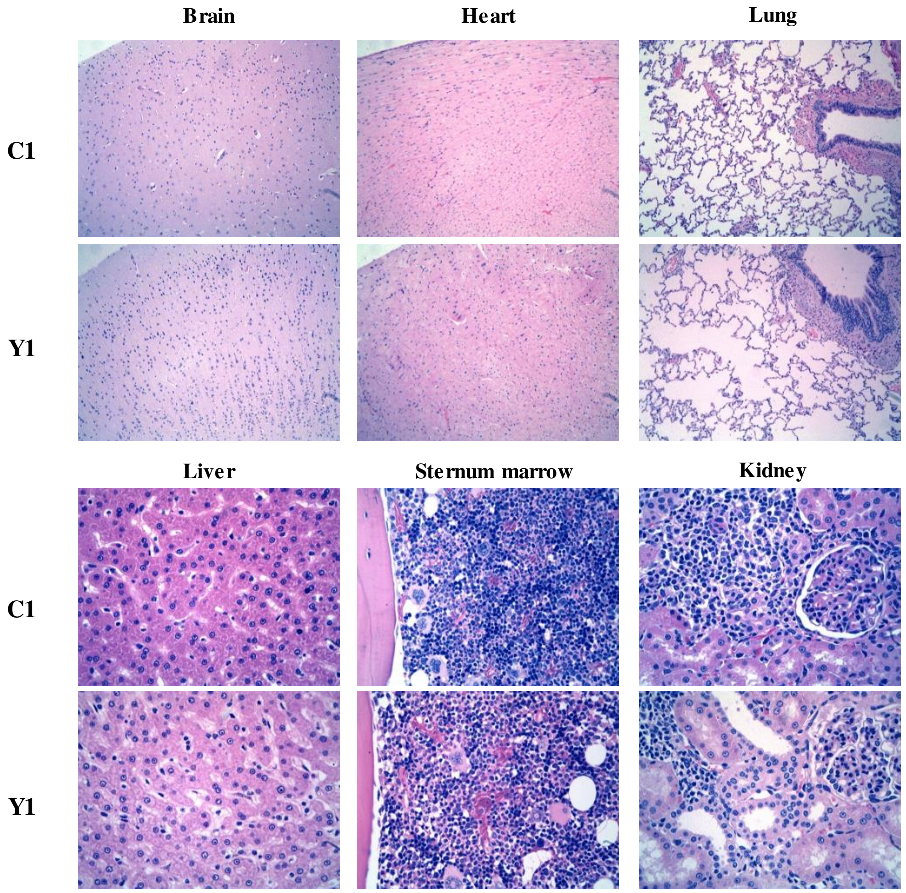

Fig. S12. Histological analysis of vital tissues from YST-OVH-treated cynomolgus monkeys and vehicle-treated cynomolgus monkeys.

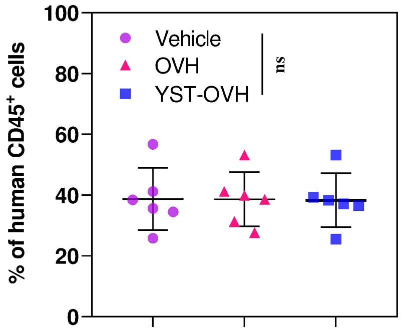

Fig. S13. The percentage of human CD45⁺ cell reconstitution in humanized mice was analyzed by flow cytometry. All values are presented as the mean ± SEM; one-way ANOVA (A).

Supplementary tables

Table S1. Experimental design of the neurovirulence study performed with rhesus macaques.

Group		Dosage （PFU/per）	Volume （mL/per）	Injection sites	Delivery speed	Number	Animal No.	
							Male	Female
1	Vehicle control	0	1	2	0.5 mL/10 min	2+3+2	CTL1	CTL2
2	YST-OVH	3.3×10^{7}	1	2		2+3+2	Rhe1-5	Rhe6-10

Table S2. Clinical symptoms of rhesus macaques in the neurovirulence study treated by intrathalamic injection of YST-OVH.

	Animal No.	Changes in responsiveness	Ruffled fur	Diarrhea	Eclampsia	Epilepsy	Tremor	Ataxia	Anorexia	Nystagmus	Shiver	Lethargy	Emesis	Hemiplegia
	Control: Saline													
Male	CTL1	-	-	-	-	-	-	-	-	-	-	-	-	-
Female	CTL2	-	-	-	-	-	-	-	-	-	-	-	-	-
	YST-OVH													
Male	Rhe1	-	-	-	-	-	-	-	-	-	-	-	-	-
	Rhe2	-	-	+	-	-	-	-	-	-	-	-	-	-
	Rhe3	-	-	-	-	-	-	-	-	-	-	-	-	-
	Rhe4	-	-	-	-	-	-	-	-	-	-	-	-	-
	Rhe5	-	-	-	-	-	-	-	-	-	-	-	-	-
Female	Rhe6	-	-	+	-	-	-	-	-	-	-	-	-	-
	Rhe7	-	-	-	-	-	-	-	-	-	-	-	-	-
	Rhe8	-	-	-	-	-	-	-	-	-	-	-	-	-
	Rhe9	-	-	-	-	-	-	-	-	-	-	-	-	-
	Rhe10	-	-	-	-	-	-	-	-	-	-	-	-	-

- No symptoms; + Slight symptoms.

Table S3. Experimental design of the toxicity study performed with cynomolgus monkeys.

Group		Dosage （PFU/per）	Volume （mL/per）	Injection times in total	Number	Animal No.	
						Male	Female
1	Vehicle control	0	3	15	5+5	C1-C5	C6-C10
2	YST-OVH	2.4×10^{8}	3	15	5+5	Y1-Y5	Y6-Y10

Table S4. Clinical symptoms of cynomolgus monkeys in the safety study treated by intravenous injection of YST-OVH.

		Animal No.	Ruffled fur	Diarrhea	Necrosis	Scurf	Hunched posture	Ataxia	Anorexia	Changes in responsiveness	Asthenia	Lethargy	Hypoexcitability
0 PFU		Control : Saline											
	Male	C1	-	-	-	-	-	-	-	-	-	-	-
		C2	-	-	-	-	-	-	-	-	-	-	-
		C3	-	-	-	-	-	-	-	-	-	-	-
		C4	-	-	-	-	-	-	-	-	-	-	-
		C5	-	+	-	-	-	-	-	-	-	-	-
	Female	C6	-	-	-	-	-	-	-	-	-	-	-
		C7	-	-	-	-	-	-	-	-	-	-	-
		C8	+	-	-	-	-	-	-	-	-	-	-
		C9	-	-	-	-	-	-	-	-	-	-	-
		C10	-	-	-	-	-	-	-	-	-	-	-
2.4×10^{8} PFU		YST-OVH											
	Male	Y1	-	+	-	-	-	-	-	-	-	-	-
		Y2	-	-	-	-	-	-	-	-	-	-	-
		Y3	-	-	-	-	-	-	-	-	-	-	-
		Y4	-	-	-	-	-	-	-	-	-	-	-
		Y5	-	-	-	-	-	-	-	-	-	-	-
	Female	Y6	+	-	-	-	-	-	-	-	-	-	-
		Y7	-	-	-	-	-	-	-	-	-	-	-
		Y8	+	-	-	-	-	-	-	-	-	-	-
		Y9	-	-	-	-	-	-	-	-	-	-	-
		Y10	-	-	-	-	-	-	-	-	-	-	-

- No symptoms; + Slight symptoms.

Table S5. Antibodies used for time-of-flight mass cytometry analysis.

No.	Label	Target	Clone	CAT#	Company	Dilution	Total Clustering	T cell Clustering
1	141Pr	Ly-6G	1A8	3141008B	Fludigm	1:200	√	
2	142Nd	CD11c	N418	3142003B	Fludigm	1:100	√	
3	143Nd	GITR	DTA1	3143019B	Fludigm	1:125	√	√
4	144Nd	CD127	A7R34	BE0065	Home-made<br>BioXCell	1:200	√	√
5	145Nd	CD69	H1.2F3	3145005B	Fludigm	1:150		√
6	146Nd	F4/80	BM8	3146008B	Fludigm	1:200	√	
7	147Sm	CD4	GK1.5	BE0003-1	Home-made<br>BioXCell	1:200	√	√
8	148Nd	CD11b	M1/70	3148003B	Fludigm	1:550	√	
9	149Sm	CD160	7H1	143002	Home-made<br>Biolegend	1:100		√
10	150Nd	CD25	3C7	3150002B	Fludigm	1:125		√
11	151Eu	ICOS	C398.4A	3151020B	Fludigm	1:200		√
12	152Sm	CD3ε	145-2C11	BE0001-1	Home-Made<br>BioXCell	1:300	√	√
13	153Eu	PD-L1	10F.9G2	3153016B	Fludigm	1:200		
14	154Sm	CTLA-4	UC10-4B9	3154008B	Fludigm	1:200	√	√
15	155Gd	CD44	IM7	BE0039	Home-made<br>BioXCell	1:300	√	√
16	156Gd	CD14	Sa14-2	3156009B	Fludigm	1:300	√	
17	158Gd	Foxp3	FJK-16s	3158003A	Fludigm	1:200	√	√
18	159Tb	TCRγδ	GL3	3159012B	Fludigm	1:150	√	
19	160Gd	CD62L	MEL-14	3160008B	Fludigm	1:300	√	√
20	161Dy	Tbet	4B10	3161014B	Fludigm	1:125	√	√
21	162Dy	Tim-3	RMT3-23	3162029B	Fludigm	1:125	√	√
22	163Dy	BCL-6	K112-91	3163012B	Fludigm	1:200		

23	164Dy	TGFβ	TW7-16B4	3164014B	Fludigm	1:200		√
24	165Ho	IFNγ	XMG1.2	3165003B	Fludigm	1:100		√
25	166Er	BTLA	6A6	BE0132	Home-made<br>BioXCell	1:200		√
26	167Er	Gata3	TWAJ	3167007A	Fludigm	1:200	√	√
27	168Er	CD8	53-6.7	BP0004-1	Home-Made<br>BioXCell	1:150	√	√
28	169Tm	TCRβ	H57-597	3169002B	Fludigm	1:300	√	√
29	170Er	NK1.1	PK136	3170002B	Fludigm	1:150	√	
30	171Yb	CD80	16-10A1	3171008B	Fludigm	1:100	√	√
31	172Yb	CD86	GL1	3172016B	Fludigm	1:100	√	√
32	173Yb	Ly6/C	HK1.4	BE0284	Home-Made<br>BioXCell	1:200	√	√
33	174Yb	LAG-3	C9B7W	3174019B	Fludigm	1:125	√	√
34	175Lu	PD-1	EH12.2H7	3175008B	Fludigm	1:300	√	√
35	176Yb	B220	RA3.3A1/6.1 (TIB-146)	BE0067	Home-Made<br>BioXCell	1:150	√	
36	209Bi	MHC class II	M5/114.15.2	3209006B	Fludigm	1:150	√	
37	89Y	CD45	30-F11	3089005B	Fludigm	1:200		
38	194Pt	KLRG1	2F1 (RUO)	562190	Home-Made<br>BD	1:150		√
39	Rh103	Live		201103A	Fludigm	1:500		
40	191/193Pt	DNA		201192B	Fludigm	1:1000		
